# Depressive Symptoms are Associated with More Negative Global Metacognitive Biases in Combat Veterans, and Biases Covary with Symptom Changes over Time

**DOI:** 10.1155/2023/2925551

**Published:** 2023-04-17

**Authors:** Sam Agnoli, Agnieszka Zuberer, Melanni Nanni-Zepeda, Regina E. McGlinchey, William P. Milberg, Michael Esterman, Joseph DeGutis

**Affiliations:** ^1^Translational Research Center for TBI and Stress Disorders (TRACTS) and Geriatric Research, Education and Clinical Center (GRECC), VA Boston Health Care System, Boston, MA, USA; ^2^Boston Attention and Learning Lab, VA Boston Health Care System, Boston, MA, USA; ^3^Department for Psychiatry and Psychotherapy, University Hospital Tübingen, Tübingen, Germany; ^4^Department of Psychiatry and Psychotherapy, Jena University Hospital, Jena, Germany; ^5^Department of Psychiatry, Harvard Medical School, Boston, MA, USA; ^6^National Center for PTSD, VA Boston Health Care System, Boston, MA, USA; ^7^Department of Psychiatry, Boston University School of Medicine, Boston, MA, USA

## Abstract

Metacognitive awareness, insight into one's abilities, is thought to be disrupted in depression and posttraumatic stress disorder (PTSD), with more negative metacognitive biases and reduced awareness, respectively. However, studies have rarely examined global aspects of metacognitive awareness in depression and PTSD, instead using task-specific measures. In 467 trauma-exposed post-9/11 veterans, we administered assessments of PTSD and depression, self-report and objective measures of cognitive functioning (neuropsychological battery of memory, attention, and executive function measures), and self-report and objective measures of general health (index of five cardiometabolic factors). We used self-report/objective correlations to measure metacognitive sensitivity and 'self-report minus objective' scores to measure bias. We also examined associations between changes in metacognitive awareness and changes in PTSD and depression over time. Metacognitive sensitivity was comparable between individuals with and without PTSD and depression. However, metacognitive bias was significantly more negative in those with greater depressive symptoms (i.e., underconfidence) across cognition and health, independent of PTSD symptoms. Notably, metacognitive bias changes covaried with depressive and PTSD symptom changes two years later. This shows that, in trauma-exposed veterans, negative metacognitive biases are specifically related to depressive symptoms and these biases may be relatively domain general. Further, our longitudinal results suggest that, rather than being a stable trait, metacognitive biases change with PTSD/depressive symptoms over time.

## 1. Introduction

Metacognition refers to the self-reflective activity of monitoring, planning, interpreting, and regulating the contents of information processing [1]. One important aspect of metacognition is awareness of one's traits, abilities, or performance. This awareness has two components: metacognitive sensitivity (i.e., accuracy) refers to a person's capacity to correctly evaluate themself, whereas metacognitive bias measures the difference between subjective and objective traits, abilities, or performance (i.e., tendency to under- vs. over-estimate, see [2]). Metacognitive awareness is posited to be impaired in depression, where pervasive negative self-evaluations are frequent [3, 4], and in PTSD, where increased autonomic arousal and deficits in cognitive control may impair awareness [5, 6]. However, studies examining metacognition in depression and PTSD have primarily measured distress-related metacognitive beliefs using self-report questionnaires (e.g., I think my worrying is dangerous; for a review, see [7]), and far fewer have examined more general metacognitive awareness. Further, studies examining metacognitive awareness in PTSD and depression have typically measured task-specific trial-by-trial confidence (e.g., How confident are you that you got that memory trial correct? [8]; for exceptions, see [9, 10]). Recently, researchers studying metacognition in clinical populations have recommended approaches to metacognitive awareness that are “global” rather than task-specific, by measuring domain-general self-beliefs and abilities [11]. This more global approach was applied to measure metacognition in the current study.

### 1.1. Metacognitive Awareness in Depression

Though depression is often associated with negative self-evaluations, evidence for metacognitive awareness deficits has been mixed. Most studies in this area have compared participants' performance on objective tasks to their subjective judgments of performance, both trial-by-trial and after a block of trials [12], allowing researchers to quantify metacognitive sensitivity and bias. Studies have observed a negative metacognitive bias across both subclinical and clinical depression with some inconsistencies, whereas studies have not generally observed metacognitive sensitivity deficits (for a review, see [12]). For example, in two large online experiments, an anxious-depressive dimension was associated with systematically negative biases (i.e., underconfidence) in both a perceptual decision task, where participants had to discern which of the two boxes had more dots, and a general knowledge task, where individuals had to discern a correct fact between two choices, despite a lack of impairment in objective performance [13]. In two studies using the same perceptual decision-making task, depressive symptoms were associated with a more negative bias and an enhanced metacognitive sensitivity [14], while there were no differences reported in metacognitive bias or sensitivity between those with and without depression in another study [15]. Other studies comparing depressed and control groups have observed negative metacognitive biases in tasks including object recognition, general knowledge, facial emotion recognition, line-length perceptual judgment [16], adjective recognition [17], the Stroop task [18], and verbal memory performance [19]. A prominent model explaining dysfunctional metacognitive awareness in depression implicates patterns of negative thinking, such as attending to negatively valenced material [20] or the increased salience of negatively valenced memories [21], that become activated during negative moods [22, 23]. These negative biases in perception and memory are resilient to feedback and inform more negative global self-performance estimates [4, 24, 25]).

Despite the evidence for depression-related negative metacognitive biases in trial-by-trial tasks, several weak or nonsignificant depression/metacognitive bias associations have been reported, including working memory (digit span backwards, rho = −0.10) and executive functioning (Wisconsin Card Sorting Task (WCST), rho = −0.04, [26]). One explanation for these inconsistent findings in trial-by-trial metacognitive biases is due to the diversity of cognitive tasks. While indexing metacognitive awareness in an isolated domain, specific cognitive tasks (e.g., WCST, [26]) may not generalize to global self-beliefs about cognitive abilities. Further, global self-beliefs such as self-esteem have been more directly related to subjective and functional experiences in depression [3, 11]. One recent study found significant associations between negative global metacognitive biases and depressive symptoms in adolescents and emerging adults by comparing confidence ratings on two perceptual tasks with the Rosenberg Self-Esteem Scale [10]. These findings are consistent with recent results from Hoven et al. [9] in a general population showing greater negative global metacognitive bias in those with more anxious-depressive symptoms. Overall, metacognitive sensitivity has not shown to be impaired in depression, while studies examining trial-by-trial metacognitive biases have been mixed, though generally observe depression-related impairments. However, few studies have examined more global aspects of metacognitive biases.

### 1.2. Metacognitive Awareness in PTSD

Compared to depression, studies examining metacognitive awareness in PTSD are relatively scarce, with most PTSD metacognition studies focusing on participants' beliefs about their trauma (e.g., hyperresponsibility and a belief about being irreparably damaged by trauma; [27]). With regard to metacognitive awareness, even after controlling for depression, hyperarousal symptoms have been associated with metacognitive deficits in PTSD on the Metacognition Assessment Scale. This scale measures a clinician's rating of a patient's awareness of illness, cognitive insight, and memory accuracy [28]. One explanation for this association is that PTSD-related increases in autonomic arousal impair processes necessary for successful self-reflection (e.g., inhibitory control; [5, 29]). Additionally, Sacher et al. [8] measured trial-by-trial confidence in episodic memory performance and found that individuals with PTSD had worse awareness of their memory deficits and more negative bias, though they did not control for depression. Studies of metacognitive therapy as developed by Wells and Sembi [30] seek to correct dysfunctional beliefs about an individual's trauma and have been shown to be effective in decreasing PTSD symptoms ([31]; see also [32]). Metacognitive therapy offers an alternative to direct trauma confrontations in prolonged exposure therapy or cognitive behavior therapy (CBT), such as by practicing detached mindfulness through the abandonment of threat monitoring; in contrast, CBT discusses cognitive content, rather than cognitive processes, and corrects the metacognitive evaluations of this content [33].

### 1.3. The Current Study

Our goal was to extend the previous literature in several important ways. First, instead of taking a task-specific approach, the current study measured global metacognitive awareness by contrasting an objective cognitive battery and five physical health factors with self-reported cognition and health, respectively. This is one of the first studies to examine how global metacognitive awareness, which may be particularly clinically and functionally meaningful, is associated with depression (see [10]), the first to do so in PTSD, and the first to measure metacognitive awareness using a domain-general battery of objective cognitive tasks. Second, by repeating our assessment battery approximately two years later in a subset of participants, the current study is the first to measure how longitudinal changes in metacognitive awareness are related to changes in depressive and PTSD symptoms. This can begin to address the critical question of whether metacognitive awareness deficits are a stable trait or rather covary with depressive and PTSD symptoms. Finally, to develop a better mechanistic understanding of the relative importance of PTSD and depressive symptoms to metacognitive awareness deficits, mediation analyses of the observed effects were performed.

To address these questions, a large group of 467 trauma-exposed post-9/11 veterans with a range of depressive and PTSD symptoms were recruited and tested. Depression and PTSD are common sequelae of trauma exposure that are frequently comorbid in veterans (see [34, 35]). Relevant to metacognitive awareness, while 72% of post-9/11 veterans report moderate to very severe cognitive impairment [36], evidence suggests that objective cognitive deficits are milder and less frequently observed (e.g., 35% of post-9/11 veterans have shown to have DSM-5-defined mild cognitive dysfunction; [37]), suggesting a potential negative metacognitive bias. In the current study, to better characterize metacognitive awareness in both sensitivity and bias, objective cognition on a validated battery of neuropsychological tasks was contrasted with subjective cognitive functioning within the cognition subscale of the World Health Organization Disability Assessment Schedule-II (WHODAS II). In addition, metacognitive health awareness was measured through contrasting general subjective health with validated objective indices of cardiometabolic health (e.g., blood pressure, cholesterol, and waist circumference; see Table S1). Finally, in 267 veterans who were reassessed approximately two years later, associations between changes in metacognitive awareness and changes in PTSD and depressive symptoms over time were examined.

## 2. Transparency and Openness

Data and syntax can be accessed pending approval from the VA Boston Health Care System IRB. Please contact Dr. Joseph DeGutis at degutis@wjh.harvard.edu. The current study is part of an ongoing longitudinal study [38]. We report how we determined our sample size, all data exclusions, manipulations, and measures in the study. The VA Boston Healthcare System IRB approved this study (#2354, Translational Research Center for Traumatic Brain Injury and Stress Disorders: Human Characterization Core B), written consent was obtained from all participants, and research was conducted in accordance with the Declaration of Helsinki.

## 3. Methods

Participants were drawn from a pool of 813 post-9/11 combat-deployed veterans recruited into the Translational Research Center for TBI and Stress Disorders (TRACTS; for a more in-depth description of the sample and measures, see [38]) who participated in data collection at time 1 and time 2 visits (e.g., ~2 years later) between the years 2010 and 2019. The TRACTS exclusionary criteria excluded participants who had a history of neurological/physical impairments (*n* = 5), moderate to severe traumatic brain injury (*n* = 38), or psychiatric disorders (*n* = 9), including bipolar disorder and/or suicidal/homicidal ideation requiring crisis intervention at either assessment. Additional participants were removed due to evidence of reduced effort on the Medical Symptom Validity Test ([39], *n* = 67) at either time point. A total of 46 participants who did not complete the WHODAS II were also excluded, as well individuals not reporting any significant interference in daily life on the WHODAS II to remove ceiling effects (*n* = 177, similar to our recent paper [40]). Notably, all key analyses replicated when including individuals that reported no significant interference in daily life (see Supplementary Materials (available here)). Participants may have been excluded for more than one reason. This left a final sample of 467 participants at time 1 and 267 at time 2.

### 3.1. Clinical Measures

PTSD diagnosis and severity were assessed using the clinician-administered PTSD scale for the DSM-IV (CAPS-IV, [41]). The CAPS-IV is a clinician-administered interview corresponding to the DSM-IV symptoms for PTSD with excellent reliability (test-retest reliability of 0.89, [42]). The current CAPS score was used as a summation of all PTSD symptoms, with total scores ranging from 0 to 136. The depression subscale from the Depression, Anxiety and Stress Scale (DASS) was used to measure continuous depressive symptoms, with total scores ranging from 0 to 42 [43]. Diagnostic measures from the Structured Clinical Interview for DSM-IV were used to measure a current depression diagnosis, as well as anxiety disorder, and substance use disorder (SCID-I/NP; [44]). Please note that while the DSM-IV measures all mood disorder, in the current sample >95% of those with a mood disorder had major depressive disorder. CAPS-IV and SCID-I/NP were kept for continuity across time points. Pain was assessed using the McGill Pain Questionnaire (MPQ; [45]), sleep was assessed using the Pittsburgh Sleep Quality Index (PSQI; [46]), and traumatic brain injury was assessed using the Boston Assessment of Traumatic Brain Injury-Lifetime [47].

### 3.2. Subjective Cognitive Functioning

The World Health Organization Disability Assessment Schedule II (WHODAS II, [48]) is a validated self-reported functional outcome measure that has been highly recommended to assess functioning in trauma-exposed post-9/11 veterans [49]. The WHODAS II is a 42-item measure with six subscales: understanding and communicating, getting around, self-care, getting along with people, life activities, and participation in society. We focused our analyses on the understanding and communicating subscale, which ranged from 0 to 100, with higher scores indicating greater disability in the cognitive domain. The subscale asked 6 questions such as “in the past 30 days, how much difficulty did you have in analyzing and finding solutions to problems in day to day life?” (see Table S1). Self-reported cognition was reverse scored in analyses, such that higher scores represent better self-reported functioning for ease of interpretation and to be consistent with objective cognition and metacognitive bias measures. After participants report their functional difficulties across the six subscales, participants are separately asked to indicate how much their functioning interferes with their daily life on a scale of 0 (none) to 4 (extreme). The final sample included participants with overall interference scores greater than 0 to avoid issues with skewness/ceiling effects [40], though analyses of interest were replicated with all veterans in the Supplementary Materials.

### 3.3. Objective Cognitive Functioning

In order to measure objective cognitive functioning, a global cognitive composite was created as an average of the standardized age-adjusted *z-*scores for three cognitive domains: executive functions, memory, and attention (see [37]). The executive function composite included the Delis-Kaplan Executive Function System (D-KEFS) Trail Making Test Number/Letter Switching subtest, i.e., Trails B, as a measure of working memory/switching [50], the D-KEFS letter fluency as a mixed executive function measure of working memory/switching (FAS category switching and letter fluency subtests, [50]), the D-KEFS Color-Word Test (i.e., the Stroop test) as a measure of inhibitory control, the CANTAB Intra-Extra Dimensional Set Shift Task (number of stages completed) as a measure of task-switching [51], and the Auditory Consonant Trigrams as a measure of working memory (ACT, [52]). The ACT was not administered to all participants at time 2; therefore, within-group changes were not examined with this measure. Verbal learning and memory were measured using the California Verbal Learning Test-Second Edition [53]. The verbal memory composite score measures encoding, recall, and recognition and consists of the mean age-adjusted *z*-scores of total learning, short-delay free recall, long-delay free recall, and long-delay recognition hits (see [37]). The attention composite included the Test of Variables Attention [54], the Digit Span Forward trials (WAIS-IV; [55]), and the Trail Number, Sequencing subtest [50], i.e., Trails A.

### 3.4. Calculating Global Metacognitive Sensitivity and Bias

Global metacognitive sensitivity, i.e., accuracy, was calculated using nonparametric Spearman's correlations between self-reported and objective cognition measures at the group level. In calculating the global metacognitive bias score, self-reported cognition (reverse scored so that higher values equate to higher subjective ability) in the WHODAS II was subtracted from the global objective cognition score as averaged across executive function, memory, and attention tasks within an individual. Bias, i.e., calibration or confidence, is usually calculated as the difference between self-reported cognitive abilities and objective task performance [12]. Scores below 0 refer to a negative metacognitive bias relative to the sample, such that an individual reports more self-reported cognitive deficits than are objectively measured. Self-reported cognition was *z*-scored within the total veteran sample in lieu of appropriate normative data, while objective cognition was calculated based on normative data [37]. The objective cognition scores were then *z*-scored within the sample so that the mean and SD of both objective and self-reported measures were 0 and 1, respectively. Because of this and dissimilarities in self-reported and objective cognition distributions, the difference score represents a relative value. For example, an individual with balanced metacognitive bias (i.e., self-reported-objective cognition = 0) should not be interpreted as having no metacognitive bias, but rather a lack of bias within the relative distribution of deployed post-9/11 veterans in the sample.

### 3.5. Self-Reported, Objective, and Metacognitive Health

Metacognitive awareness of health was measured by comparing self-reported and objective measures of physical health. Metacognitive health bias and sensitivity scores were calculated identically to their cognition counterparts. For subjective health, participants rated a single item, “How would you rate your general health?”, on a Likert scale ranging from 1, “excellent,” to 4, “poor.” Objective health was assessed using comprehensive physical and physiological measures of five cardiometabolic syndrome risk factors: obesity (waist circumference), triglycerides, high density lipoprotein, blood pressure, and glucose levels (for more information, see [38, 56]). The dependent variable was the total cardiometabolic risk factor score for each participant, ranging from 0 to 5, representing the total number of elevated/abnormal risk factors. The sum of the risk factors was used because it represents a unified construct (cardiometabolic health) and has been used in previous studies (e.g., [57]). For consistency with cognition measures, both self-reported and objective health were reverse scored such that higher scores represent better health.

### 3.6. Longitudinal Analyses

Because not all participants returned for time 2, longitudinal analyses were performed in a reduced sample (*n* = 267). Global metacognitive sensitivity was computed as before and compared between time points. In calculating a longitudinal difference score in global metacognitive bias and clinical measures of interest, time 2 scores were subtracted from time 1. Changes in global metacognitive bias and self-reported and objective cognition were then associated using Spearman's correlations with changes in clinical measures to see if they tracked with symptom changes and to further explore specificity. Finally, in order to more completely characterize changes in metacognitive bias scores, a reduced sample was analyzed after grouping by changes in diagnoses of a current PTSD or depression disorder between time points: improved depression, worsened depression, improved PTSD, and worsened PTSD. For example, the improved depression group had depression at time 1 and no longer met criteria for depression at time 2, while the worsened group had depression at time 1 and subsequently developed depression at time 2 (full analyses available in the Supplementary Materials). A 2 × 2 repeated measures ANOVA was run, with time 1/time 2 metacognitive bias and group (e.g., no depression diagnosis to depression diagnosis vs. depression diagnosis to no depression diagnosis) as factors.

### 3.7. Sample Size Justification

The current study is part of an ongoing longitudinal study that has consistently found significant associations between PTSD/depressive symptoms and self-reported and objective cognitive functioning [37] as well as inhibitory control and PTSD [29, 40]. Previous studies have found significant associations between objective performance on cognitive tasks and functional behaviors (*n* = 489, [9]; *n* = 57, [10]). Relevant to the current investigation, a recent study found significant associations between greater depressive symptoms and more negative metacognitive bias by comparing two perceptual tasks with global self-esteem estimates (a similar measure to our subjective cognition score), finding a small effect size of *β* = 0.117 [9]. Considering that Hoven et al. [9] used two brief tasks and we used a considerably more reliable 3-hour objective cognitive battery, we estimate that we would have at least a 25% larger effect size (*β* = 0.146). Using a *α* = 0.05 and 1 − *β* = 0.80, our study should require 362 participants to adequately detect these associations. Therefore, given our sample size (*n* = 467), we estimate that we will have enough power to detect an effect size approximately the same as the previous literature (*β* ≥ 0.146, where a small effect is 0.2).

## 4. Results

### 4.1. Demographics and Clinical Characteristics

As can be seen in Table 1, the sample of 467 post-9/11 veterans was representative of the US military, with 90% male, 72% white, a mean age of 34.61 years (*SD* = 8.95), and 14.18 years of education (*SD* = 2.15). Of the 164 veterans with a diagnosis of depression and 299 veterans with PTSD, 145 had comorbid PTSD and depression, while 148 had neither. Individuals with comorbid disorders had higher PTSD symptom severity (*M* = 78.55, *SD* = 21.65) than those with PTSD alone (*M* =62.71, *SD* = 15.74; *t* = 8.23, *p* < 0.001) and numerically higher depressive symptoms (*M* = 19.56, *SD* = 9.92) than those only with depression (*M* = 15.37, *SD* = 9.84; *t* = 1.73, *p* = 0.085). Notably, PTSD and depressive symptoms were robustly correlated (*ρ* = 0.53, *p* < 0.001).

### 4.2. Self-Reported and Objective Cognition and Health

Before examining measures of metacognitive awareness, self-reported and objective performance across cognition and health were first examined. With regard to self-reported cognition, a between-group ANOVA revealed significant differences, with the greatest self-reported deficits in those with PTSD and depression diagnoses, then depression only, PTSD only, and the least in those with neither disorder (*F*(3,462) = 24.07, *p* < 0.001; see Table 2). In contrast, objective cognition did not significantly differ between the groups (*F*(3,462) = 1.61, *p* = .185). Further, an omnibus ANOVA was run comparing our three largest groups (removing depression only), and no significant differences in objective cognition were found (*F*(2,437) = 2.43, *p* = 0.089). Consistent with these results, depressive and PTSD symptoms had moderate to strong associations with self-reported cognitive deficits (depression: *ρ* = −0.56, *p* < 0.001; PTSD: *ρ* = −0.47, *p* < 0.001), though weaker but still significant associations with objective global cognitive deficits (depression: *ρ* = −0.18, *p* < 0.001; PTSD: *ρ* = −0.20, *p* < 0.001).

A similar pattern when examining health was found. A between-groups ANOVA revealed significant differences, with the greatest self-reported deficits in those with PTSD and depression diagnoses, then depression only, PTSD only, and the least in those with neither disorder (*F*(3,462) = 7.54, *p* < 0.001; see Table 2). In contrast, objective health did not significantly differ between the groups (*F*(3,462) = 0.78, *p* = .504). Further, depressive and PTSD symptoms were significantly associated with self-reported health (depression: *ρ* = −0.30, *p* < 0.001; PTSD: *ρ* = −0.22, *p* < 0.001) but were not significantly associated with objective health measures (depression: *ρ* = −0.09, *p* = 0.061; PTSD: *ρ* = −0.07, *p* = 0.128; see Table S5).

### 4.3. Global Metacognitive Awareness

#### 4.3.1. Metacognitive Sensitivity

**Figure 1:**
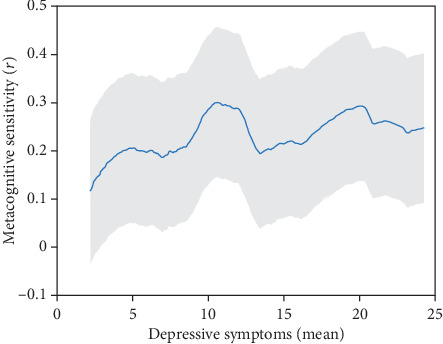
Metacognitive sensitivity by DASS depression symptom severity. *Note*. Metacognitive sensitivity was calculated as the correlation between self-reported functioning on the WHODAS II and objective cognitive functioning on a battery of cognitive tasks. Sensitivity was graphed using a continuous sliding window by calculating 100 iterations of sensitivity at respective depressive symptoms using bins of *n* = 150.

Next, the study's measures of interest were examined: global metacognitive sensitivity and bias in the domains of cognition and health. Metacognitive sensitivity was first assessed using Spearman's correlations between self-reported and objective scores. Across the sample, veterans had significant metacognitive sensitivity across cognition (*ρ* = 0.26, *p* < 0.001) and health (*ρ* = 0.17, *p* < 0.001). To determine if this differed based on PTSD/depression diagnosis, an overall comparison of associations between the four groups (PTSD and depression, depression only, PTSD only, and neither disorder) was run. There were no differences between groups (*F*(3,462) = 2.08, *p* = 0.556), though metacognitive sensitivity was consistently above 0 (see Figure 1 and Table 2; for metacognitive health sensitivity in depression, see Figure S1; for metacognitive sensitivity in PTSD, see Figure S2).

#### 4.3.2. Metacognitive Bias

**Figure 2:**
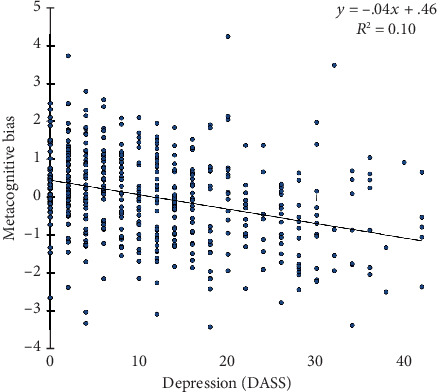
Metacognitive bias in cognition varies with depressive symptoms. *Note*. Higher metacognitive bias indicates more positive judgements of one's cognitive abilities relative to our sample. Greater DASS depression scores indicate more severe depressive symptoms.

Next, metacognitive biases in awareness (i.e., self-reported minus objective performance) in cognition and health and their relationships to depression and PTSD symptoms were examined. Anxiety was not prioritized in the current study because few veterans had a current non-PTSD anxiety disorder (*n* = 93) on the SCID-I/NP, and anxiety symptoms were strongly correlated with depressive symptoms (*ρ* = 0.65, *p* < 0.001). For cognition, greater negative metacognitive bias was significantly associated with increased depressive (*ρ* = −0.32, *p* < 0.001) and PTSD (*ρ* = −0.23, *p* < 0.001) symptoms. Only depressive symptoms were a significant predictor of metacognitive bias when PTSD and depressive symptoms were entered into a regression (depression: *β* = −0.27, *p* < 0.001; PTSD: *β* = −0.08, *p* = 0.11; see Figure 2). We further ran correlations between DSM-IV PTSD symptom clusters and metacognitive bias and observed similar results across PTSD subscales (see Supplementary Material). However, depressive symptoms mediated PTSD in predicting metacognitive bias, such that worse depressive symptoms partially explained the relationship between PTSD symptoms and more negative metacognitive bias (*β* = −0.17, 95% CI: -0.25, -0.09) with a direct effect of PTSD no longer being significant (*β* = −0.10, *p* = 0.110). In contrast, PTSD did not mediate depression's association with metacognitive bias (indirect *β* = −0.05, 95% CI: -0.12, 0.01; direct effect of depression *β* = −0.325, *p* < 0.001).

**Figure 3:**
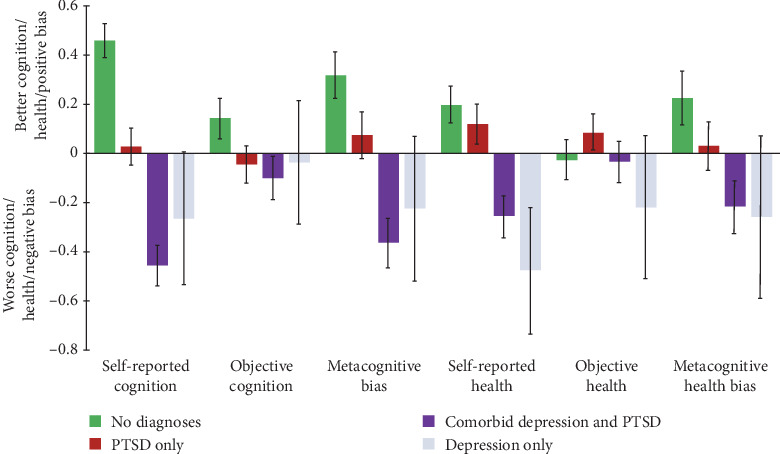
Self-reported, objective, and metacognitive bias measures across cognition and health in PTSD and depression diagnostic groups. *Note*. In the interest of showing all of the data, we included veterans with only depression (*N* = 19) on the rightmost bar of each measure in a faded blue. For the sake of graphical interpretation, self-reported cognition, self-reported health, and objective health were reverse scored, such that positive values of all measures represent improved functioning relative to the sample. Error bars represent standard error.

Metacognitive bias differences in cognition were further characterized by comparing diagnostic groups (PTSD and depression, PTSD only, depression only, and neither disorder; see Figure 3). An omnibus ANOVA revealed significant differences between groups (*F*(3,462) = 8.56, *p* < 0.001; see Table 2). No differences were found between those with only a PTSD diagnosis (*M* = 0.07, *SD* = 1.16) and those with neither disorder (*M* = 0.32, *SD* = 1.16; *t* = 1.82, *p* = 0.070). Additionally, only a trend of a difference emerged between those with depression only (*M* = −0.23, *SD* = 1.29) and those with neither disorder (*t* = 1.91, *p* = 0.059), likely due to limited power (depression only *n* = 19). However, a significant difference between those with comorbid PTSD and depression (*M* = −0.36, *SD* = 1.19) and those with only a PTSD diagnosis (*M* = 0.07, *SD* = 1.16; *t* = 3.19, *p* = 0.002, *q* = 0.006) was found, such that having comorbid PTSD and depression diagnoses was associated with a more negative appraisal of their cognitive abilities than veterans with a PTSD diagnosis alone.

Next, metacognitive biases in health were examined, which showed a very similar pattern to cognition. Across the sample, a more negative metacognitive health bias was associated with greater depressive symptoms (*ρ* = −0.12, *p* = 0.011; see Figure S3), though not PTSD symptoms (*ρ* = −0.07, *p* = 0.125). An omnibus ANOVA revealed significant differences in metacognitive health bias between diagnostic groups (PTSD and depression, PTSD only, depression only, and neither disorder, *F*(3,462) = 2.36, *p* = 0.022; see Table 2). Veterans with comorbid depression and PTSD displayed significantly more negative metacognitive biases (*M* = −0.22, *SD* = 1.28) compared to those without either diagnosis (*M* = 0.23, *SD* = 1.31; *t*(290) = 2.94, *p* = 0.004). In contrast, veterans with PTSD only showed a trend toward a more negative metacognitive bias compared to those with neither disorder (*M* = 0.03, *SD* = 1.21; *t*(290) = 1.82, *p* = 0.070).

### 4.4. Associations between Changes in Depression/PTSD and Changes in Metacognitive Awareness

To understand if metacognitive awareness is a stable trait or rather fluctuates with depressive and PTSD symptoms, associations between changes in metacognitive measures and changes in PTSD and depressive symptoms were examined over an approximately 2-year period. Changes in metacognitive bias from time 1 to time 2 were associated with changes in both depressive (*ρ* = −0.25, *p* < 0.001) and PTSD (*ρ* = −0.33, *p* < 0.001) symptoms, such that decreases in PTSD and depressive symptoms were associated with more positive changes in metacognitive bias. In a joint regression, the changes in PTSD symptoms and changes in depressive symptoms explained unique variance in changes in metacognitive bias (*R*^2^ = 0.12; *β* = −0.244, *p* < 0.001; *β* = −0.182, *p* = 0.004, respectively). Changes in PTSD significantly mediated the effect of changes in depressive symptoms in predicting changes in metacognitive bias, such that worse PTSD symptoms partially explained the relationship between depressive symptoms and more negative metacognitive bias (*β* = −0.11, 95% CI: -0.20, -0.04) with a direct effect of PTSD remaining significant (*β* = −0.16, *p* = 0.005). In order to more completely characterize changes in metacognition, veterans were separated into groups depending on their changes in PTSD or depression diagnoses. In general, findings in metacognitive bias and health were numerically consistent with continuous analyses, where biases became more negative when veterans developed PTSD or depression and inversely became more positive as diagnoses remitted, though these analyses lacked the power to reach significance (see Supplementary Materials and Tables S2-4).

Changes in metacognitive bias in health showed a similar trend as cognition in their relationship with both changes in depressive and PTSD symptoms but failed to reach significance (see Table 3). Global metacognitive sensitivity in cognition and health was not significantly related to changes in depression or PTSD (see Supplementary Materials (available here)). Associations between baseline clinical and demographic variables with changes in metacognitive bias measures were generally nonsignificant (see Table S7).

## 5. Discussion

The current study is one of the few to examine associations between global metacognitive awareness and depressive/PTSD symptoms and the first to examine how longitudinal changes in metacognitive awareness relate to changes in depressive/PTSD symptoms. More severe depressive and PTSD symptoms were significantly associated with more negative metacognitive biases in cognition, i.e., underconfidence (*ρ* = −0.32, -0.23, respectively), but were not associated with differences in metacognitive sensitivity. A similar pattern of more negative metacognitive bias with greater depressive/PTSD symptoms was also observed in the domain of health, indicating that metacognitive biases in depression and PTSD may be quite general. Notably, depressive symptoms partially mediated the relationship between PTSD symptoms and negative metacognitive bias in cognition but not vice versa, suggesting that depressive symptoms are driving this relationship. Longitudinal changes in metacognition were also examined, where changes in metacognitive bias in cognition were significantly associated with changes in both depressive and PTSD symptoms. This result is more consistent with metacognitive bias being closely linked with PTSD and depressive symptoms rather than being a stable precursor or risk factor for the development of depression/PTSD. Together, these findings advance models of metacognition in depression and PTSD as well as have important clinical and treatment implications.

The current results suggest that depressive and PTSD symptoms are consistently associated with a negative global metacognitive bias in awareness. In contrast to mixed results linking negative metacognitive bias to depressive symptoms in single, idiosyncratic cognitive tasks (multiple studies reporting null or positive biases, e.g., [12]), the current results indicate a robust negative bias on a more general objective cognitive measure composed of standardized neuropsychological tests. Additionally, this negative bias was present when participants made judgments about their general health. The current study's consistent and robust PTSD/depression associations with global measures of metacognitive bias contrasts mixed results with local trial-by-trial tasks and supports the potential benefit of using global measures of metacognition and their transdiagnostic relation to functioning, as some researchers have recently advocated for [9, 10]. In these studies, global measures were informed by local trial-by-trial confidence ratings [58, 59]. The current study extends these studies by contrasting a battery of objective tasks with self-reported cognitive functioning during daily activities (i.e., not specifically asking about task performance), which may capture additional daily life cognitive awareness information. These results are consistent with a recent theoretical review by Seow and colleagues [11] calling for more global, transdiagnostic approaches to metacognitive awareness, as opposed to measuring metacognitive awareness exclusively in isolated abilities, which make up the vast majority of the clinical metacognitive awareness literature. The consistency of the current study's results across both cognition and health provides preliminary evidence that there is a general global metacognitive bias associated with greater depressive and PTSD symptoms.

The results also showed that metacognitive awareness deficits were particularly associated with greater depressive symptoms. In a joint model, greater depressive symptoms, but not PTSD symptoms, explained unique variance in predicting a more negative metacognitive bias in cognition. Depressive symptoms also partially mediated the association of PTSD symptoms with metacognitive bias in cognition, while PTSD symptoms did not mediate depressive symptoms. Consistent with this, comorbid PTSD and depression were associated with significantly more negative biases in cognition than those with PTSD only, while bias scores in those with PTSD only and those with neither PTSD or depression were not significantly different. These analyses were repeated using self-reported and objective health scores and similarly observed a significant association between more negative metacognitive biases in general health and greater depressive symptoms, though not PTSD symptoms. These findings are consistent with previous research implicating negative metacognitive bias in depression (e.g., [10, 13]). Past studies have found weak or nonsignificant associations using task-specific measures (e.g., [26]) and have only recently begun to compare global and task-specific measures of metacognitive awareness (e.g., perceptual knowledge; [9, 10]). The current findings extend these studies by providing evidence for the domain-general nature of this depressive negative metacognitive bias through associating a cognitive battery spanning memory, attention, and executive functions as well as objective health measures with self-reported functioning. Further, the current results suggest that, in a trauma-exposed population, this relationship is driven by depressive rather than PTSD symptoms. Future studies would be useful to examine whether these depression-related negative biases extend to other functional domains (e.g., social skills or emotion regulation).

One reason why metacognitive bias could be more related to depressive symptoms than PTSD symptoms may be the particular tendency in depression to attend to negatively valenced material or have more readily available access to negatively valenced memories (e.g., [20, 24]). For example, one study examining emotional memory biases in an implicit learning task found that only depressive symptoms explained unique variance in a bias to remember negatively valenced adjectives in a joint model with anxiety, ADHD, and autism symptoms [21]. Related to this point, the self-reported cognition measure asked participants to think back about their cognitive abilities over the last 30 days. This long-term memory component is much greater than in trial-by-trial metacognitive awareness studies and could be another reason we found more robust effects between metacognitive bias and depression than the previous studies. Other behaviors cardinal to depression such as rumination [60] and belief inflexibility [61] may further exacerbate these memory biases as depression worsens, forming rigid, global distortions in an individual's abilities and plausibly leading to an overestimation of dysfunction in self-reported functioning.

Notably, metacognitive sensitivity was not reduced in those with depression or PTSD, suggesting that deficits are specific to biases. Metacognitive sensitivity in the current sample (e.g., diagnosis of depression and PTSD: *ρ* = 0.34) was similar in strength to studies of metacognitive awareness using local cognitive tasks (e.g., word and number recall *r* = 0.25 [62]; perceived knowledge *r* = 0.27 [63]) and comparable to metacognitive awareness measured using broad measurements of daily functioning (e.g., academic ability *r* = 0.21–0.39; vocational skills *r* = 0.19–0.36, [64]). Most abilities engender little feedback for one's self, let alone one's relative abilities compared to others. It could be that the better-than-average effect, the tendency for a person to perceive themselves as superior compared to their peers [65], may restrict the range of self-reported ability, potentially reducing metacognitive sensitivity. In contrast, individuals with depression are less susceptible to this effect [66] and may use a larger range of self-reported ability (e.g., comorbid depression and PTSD, *SD* = 19.00, and neither disorder, *SD* = 16.03). This expanded range could explain why we saw numerically greater metacognitive awareness sensitivity in those with PTSD and depression (*ρ* = 0.34) than those with neither disorder (*ρ* = 0.21). Rather than representing a scaling issue, another possibility is that numerically greater sensitivity reflects the “depressive realism” hypothesis, which argues that depressed individuals are better able to make certain judgments than nondepressed individuals [67]. The depressive realism hypothesis posits that depressed individuals would have increased metacognitive sensitivity along with relatively negative metacognitive biases, which the current study offers some support for, and is consistent with a recent meta-analysis finding a weak depressive realism effect [66].

The current study supports a model of metacognitive bias as being closely linked with depressive/PTSD symptom changes rather than being a more stable trait. Longitudinal analyses over a two-year period revealed significant associations between changes in depressive and PTSD symptoms with changes in metacognitive bias (*ρ* = −0.25, -0.33, respectively). This was driven by changes in self-reported cognition while objective cognition remained relatively stable. To our knowledge, this is the first study relating longitudinal changes in global metacognitive awareness with changes in PTSD or depressive symptoms. One potential explanation is that increases in symptoms of depression and PTSD, such as depressed mood and feelings of worthlessness, could lead to a more negative metacognitive bias across cognition and health. Alternatively, changes in metacognitive bias could lead to changes in depressive symptoms. A recent study found that metacognitive beliefs on the Metacognitions Questionnaire, a self-reported measure of metacognitive beliefs, predicted anxiety symptoms two months later [68]. Positive beliefs around rumination (i.e., that rumination is a useful coping strategy) also predicted greater depressive symptoms two months later [69]. However, it remains unclear if more global metacognitive biases precede changes in depressive symptoms.

Metacognitive bias changes co-occurring with or supporting changes in depression and PTSD symptoms have important clinical implications. For example, one core component of cognitive behavioral therapy for depression involves challenging and correcting perceived incompetence [70]. Indeed, improvements in executive functions were associated with greater treatment response from CBT in adults with depression or anxiety [71]. However, therapies specifically targeting metacognitive biases or sensitivity are rare. One approach, called metacognitive training, has been used in depression to successfully improve metacognitive sensitivity and bias (e.g., in false memories, [72]) as well as depressive symptoms generally [73]. In a nonclinical sample, another study training metacognitive memory ability by providing feedback to participants' predictions after each trial found both improved bias and sensitivity on that task ([74]; see also [75, 76]). A recent study found domain-general improvements on metacognitive bias, where feedback after completion of a perceptual discrimination task was associated with metacognitive awareness improvements on that task and another untrained recognition memory task [77]. Generally, cognitive feedback training has shown that it is possible to improve metacognitive bias and sensitivity, though additional research is needed to examine if these improvements consistently generalize to reduced depressive and PTSD symptoms and/or improved functioning in daily life.

## 6. Limitations

Though the current findings provide important insights into global metacognitive bias and accuracy, they have several limitations. Participants were combat-exposed post-9/11 veterans, which were predominantly white, male, and middle aged, and therefore may not generalize to other populations. Additionally, the self-reported cognition measure showed floor effects that necessitated exclusion of individuals without any daily life functional interference. However, the results were nearly identical when these participants were included (see Supplementary Materials (available here)). Further, normative data for American adults on the WHODAS II were not available, while the objective cognition measures were age-adjusted and *z*-transformed based on normative samples. As a result, the zero point of the metacognitive bias score was relative to the sample rather than to the general population. Another potential limitation is that objective cognition in a laboratory setting could be slightly overestimated in those with greater depressive/PTSD symptoms, where dynamics and stressors of daily functioning could further impair cognition [78, 79]. That being said, the metacognitive bias in health results were very similar to cognition, and it is unlikely that objective health measures differ between the lab and real world. Still, it could be interesting for future studies to investigate objective and self-reported abilities in more real-world settings using ecological momentary assessments [80]. Finally, while consistent relationships between metacognitive bias and depression over PTSD were found, the sample had considerable comorbidity between PTSD and depression. A sample with more individuals with depression alone would be useful to dissociate the effects of PTSD from depression.

## 7. Summary

The current study implicates depressive symptoms above and beyond PTSD symptoms in negative global metacognitive biases and suggests that changes in metacognitive bias fluctuate with depressive and PTSD symptoms two years later. While metacognitive sensitivity was unrelated to the severity of depressive and PTSD symptoms, robust associations between depressive symptoms and negative global metacognitive biases across both cognition and health were observed, attesting to the generality of this association. The current results provide evidence for the utility of more global measures of metacognitive awareness in characterizing psychiatric symptoms and their relation to symptom changes and outline future clinical targets to reduce metacognitive bias and potentially enhance outcomes in those suffering from depression and PTSD.

## Figures and Tables

**Table 1:** Time 1 demographics and clinical characteristics.

Variable	
*N*	467
Gender identity (M : F)	421 : 46
Age	34.61 (8.95)
Race	
Black	15%
White	72%
Other	13%
Education (years)	14.18 (2.15)
Estimated premorbid IQ (WTAR)	103.91 (11.63)
PTSD symptoms severity (CAPS-IV)	55.09 (26.75)
Depression symptoms severity (DASS)	12.10 (10.10)
Anxiety symptoms severity (DASS)	39.80 (24.58)
Pain severity (MPQ)	1.07 (1.96)
Overall daily life functioning (WHODAS II)	30.26 (16.48)
Self-reported cognition (WHODAS II)	30.46 (19.07)
Objective cognition	-0.10 (0.59)
Self-reported health	2.36 (0.67)
Objective health	1.40 (1.30)
Current PTSD diagnosis (%)	64%
Current depression disorder (%)	35%
Comorbid PTSD and depression disorder (%)	31%
Current anxiety disorder (%)	20%
Current substance use disorder (%)	18%
Military mTBI (%)	58%
Lifetime mTBI (%)	75%

**Table 2:** Self-reported, objective, and metacognitive awareness and sensitivity measures across cognition and health.

	Means and correlations				*F*	*p*
	No diagnosis (*n* = 147)	PTSD only (*n* = 152)	Depression only (*n* = 19)	Comorbid depression and PTSD (*n* = 141)		
Self-reported cognition	21.71 (16.03)	29.92 (17.62)	35.53 (22.45)	39.17 (19.00)	24.07	<0.001
Objective cognition	-0.02 (0.59)	-0.13 (0.55)	-0.13 (0.65)	-0.16 (0.62)	1.61	0.185
Metacognitive bias	0.32 (1.16)	0.074 (1.16)	-0.23 (1.29)	-0.36 (1.19)	8.56	<0.001
Self-reported health	2.23 (0.62)	2.28 (0.67)	2.68 (0.75)	2.53 (0.68)	7.54	<0.001
Objective health	1.44 (1.30)	1.29 (1.21)	1.68 (1.64)	1.45 (1.30)	0.78	0.504
Metacognitive health bias	0.23 (1.31)	0.03 (1.31)	-0.26 (1.44)	-0.22 (1.28)	3.26	0.022
Cognitive sensitivity	0.21⁣^∗^	0.21⁣^∗∗^	0.36	0.34⁣^∗∗^		
Health sensitivity	0.08	0.21⁣^∗∗^	0.28	0.21		

*Note*. ⁣^∗^*p* < 0.05 and ⁣^∗∗^*p* < 0.01.

**Table 3:** Pearson's correlations between T1 and T2 changes in measures of interest.

Difference scores	Self-reported cognition	Objective cognition	Metacognitive bias	Self-reported health	Objective health	Metacognitive health bias	Depression (DASS)
Objective cognition	0.00						
Metacognitive bias	0.69⁣^∗∗^	0.66⁣^∗∗^					
Self-reported health	0.08	-0.05	-0.03				
Objective health	0.02	-0.06	0.00	-0.03			
Metacognitive health bias	0.04	0.06	-0.01	0.66⁣^∗∗^	0.67⁣^∗∗^		
Depression (DASS)	-0.41⁣^∗∗^	-0.04	-0.25⁣^∗∗^	-0.14⁣^∗^	-0.07	-0.06	
PTSD (CAPS IV)	-0.38⁣^∗∗^	0.07	-0.33⁣^∗∗^	-0.17⁣^∗∗^	-0.05	-0.06	0.33⁣^∗∗^

*Note*. ⁣^∗^*p* < 0.05 and ⁣^∗∗^*p* < 0.01.

## Data Availability

The clinical data used to support the findings of this study are restricted by the VA Boston Health Care System IRB in order to protect veterans' patient privacy. Data are available by contacting Dr. Joseph DeGutis at degutis@wjh.harvard.edu for researchers who meet the criteria for access to confidential data.

## Supplementary Materials

Supplementary Materials For a detailed listing of self-reported and objective cognition measures, see Table S1. For the results of metacognitive sensitivity in health and PTSD, see Figures S1-S2. For the results of metacognitive bias in health by depressive symptoms, see Figure S3. For the changes in metacognitive sensitivity and bias across time points, see Tables S2-4. For correlations between self-reported, objective, and metacognitive bias measures, see Table S5. For the metacognitive biases' relationship with clinical and demographic variables, see Table S6. For the correlations between baseline clinical and demographic variables with changes in self-reported, objective, and metacognitive measures, see Table S7.

## References

[1] Purdon C., Clark D. A. (1999). Metacognition and obsessions. *Clinical Psychology & Psychotherapy*.

[2] Fleming S. M., Lau H. C. (2014). How to measure metacognition. *Frontiers in Human Neuroscience*.

[3] Orth U., Robins R. W. (2013). Understanding the link between low self-esteem and depression. *Current Directions in Psychological Science*.

[4] Rouault M., Dayan P., Fleming S. M. (2019). Forming global estimates of self-performance from local confidence. *Nature Communications*.

[5] Aupperle R. L., Melrose A. J., Stein M. B., Paulus M. P. (2012). Executive function and PTSD: disengaging from trauma. *Neuropharmacology*.

[6] Takarangi M. K. T., Smith R. A., Strange D., Flowe H. D. (2017). Metacognitive and metamemory beliefs in the development and maintenance of posttraumatic stress disorder. *Clinical Psychological Science*.

[7] Lenzo V., Sardella A., Martino G., Quattropani M. C. (2020). A systematic review of metacognitive beliefs in chronic medical conditions. *Frontiers in Psychology*.

[8] Sacher M., Tudorache A. C., Clarys D., Boudjarane M., Landré L., El-Hage W. (2018). Prospective and retrospective episodic metamemory in posttraumatic stress disorder. *Journal of Clinical and Experimental Neuropsychology*.

[9] Hoven M., Denys D., Rouault M., Luigjes J., van Holst R. (2022). How Do Confidence and Self-Beliefs Relate in Psychopathology: A Transdiagnostic Approach.

[10] Rouault M., Will G. J., Fleming S. M., Dolan R. J. (2022). Low self-esteem and the formation of global self-performance estimates in emerging adulthood. *Translational Psychiatry*.

[11] Seow T. X. F., Rouault M., Gillan C. M., Fleming S. M. (2021). How local and global metacognition shape mental health. *Biological Psychiatry*.

[12] Hoven M., Lebreton M., Engelmann J. B., Denys D., Luigjes J., van Holst R. J. (2019). Abnormalities of confidence in psychiatry: an overview and future perspectives. *Translational Psychiatry*.

[13] Benwell C. S. Y., Mohr G., Wallberg J., Kouadio A., Ince R. A. A. (2022). Psychiatrically relevant signatures of domain-general decision-making and metacognition in the general population. *Npj Mental Health Research*.

[14] Rouault M., Seow T., Gillan C. M., Fleming S. M. (2018). Psychiatric symptom dimensions are associated with dissociable shifts in metacognition but not task performance. *Biological Psychiatry*.

[15] Culot C., Lauwers T., Fantini-Hauwel C. (2023). Contributions of age and clinical depression to metacognitive performance. *Consciousness and Cognition*.

[16] Fu T., Koutstaal W., Fu C. H. Y., Poon L., Cleare A. J. (2005). Depression, Confidence, and Decision: Evidence against Depressive Realism. *Journal of Psychopathology and Behavioral Assessment*.

[17] Fu T. S. T., Koutstaal W., Poon L., Cleare A. J. (2012). Confidence judgment in depression and dysphoria: the depressive realism vs. negativity hypotheses. *Journal of Behavior Therapy and Experimental Psychiatry*.

[18] Drueke B., Gauggel S., Weise L., Forkmann T., Mainz V. (2022). Metacognitive judgements and abilities in patients with affective disorders. *Current Psychology*.

[19] Soderstrom N. C., Davalos D. B., Vázquez S. M. (2011). Metacognition and depressive realism: evidence for the level-of-depression account. *Cognitive Neuropsychiatry*.

[20] Marchetti I., Everaert J., Dainer-Best J., Loeys T., Beevers C. G., Koster E. H. (2018). Specificity and overlap of attention and memory biases in depression. *Journal of Affective Disorders*.

[21] Duyser F. A., Van Eijndhoven P. F. P., Bergman M. A. (2020). Negative memory bias as a transdiagnostic cognitive marker for depression symptom severity. *Journal of Affective Disorders*.

[22] Sun X., Zhu C., So S. H. W. (2017). Dysfunctional metacognition across psychopathologies: a meta-analytic review. *European Psychiatry*.

[23] Teasdale J. D., Moore R. G., Hayhurst H., Pope M., Williams S., Segal Z. V. (2002). Metacognitive awareness and prevention of relapse in depression: empirical evidence. *Journal of Consulting and Clinical Psychology*.

[24] Gotlib I., Krasnoperova E. (1998). Biased Information Processing as a Vulnerability Factor for Depression. *Behavior Therapy*.

[25] Williams L. M., Gatt J. M., Schofield P. R., Olivieri G., Peduto A., Gordon E. (2009). ‘Negativity bias’ in risk for depression and anxiety: brain-body fear circuitry correlates, 5-HTT-LPR and early life stress. *NeuroImage*.

[26] Quiles C., Prouteau A., Verdoux H. (2015). Associations between self-esteem, anxiety and depression and metacognitive awareness or metacognitive knowledge. *Psychiatry Research*.

[27] Brown L. A., Belli G. M., Asnaani A., Foa E. B. (2019). A review of the role of negative cognitions about oneself, others, and the world in the treatment of PTSD. *Cognitive Therapy and Research*.

[28] Lysaker P. H., Dimaggio G., Wickett-Curtis A. (2015). Deficits in metacognitive capacity are related to subjective distress and heightened levels of hyperarousal symptoms in adults with posttraumatic stress disorder. *Journal of Trauma & Dissociation*.

[29] DeGutis J., Esterman M., McCulloch B., Rosenblatt A., Milberg W., McGlinchey R. (2015). Posttraumatic psychological symptoms are associated with reduced inhibitory control, not general executive dysfunction. *Journal of the International Neuropsychological Society*.

[30] Wells A., Sembi S. (2004). Metacognitive therapy for PTSD: a preliminary investigation of a new brief treatment. *Journal of Behavior Therapy and Experimental Psychiatry*.

[31] Wells A., Colbear J. S. (2012). Treating posttraumatic stress disorder with metacognitive therapy: a preliminary controlled trial. *Journal of Clinical Psychology*.

[32] Brown R. L., Wood A., Carter J. D., Kannis-Dymand L. (2022). The metacognitive model of post-traumatic stress disorder and metacognitive therapy for post-traumatic stress disorder: a systematic review. *Clinical Psychology & Psychotherapy*.

[33] Simons M. (2010). Metakognitive und andere kognitiv-verhaltenstherapeutische verfahren bei posttraumatischer belastungsstörung. *Verhaltenstherapie*.

[34] Hankin C. S., Spiro A., Miller D. R. (1999). Mental disorders and mental health treatment among U.S. Department of Veterans Affairs outpatients: the veterans health study. *American Journal of Psychiatry*.

[35] Nichter B., Norman S., Haller M., Pietrzak R. H. (2019). Psychological burden of PTSD, depression, and their comorbidity in the U.S. veteran population: suicidality, functioning, and service utilization. *Journal of Affective Disorders*.

[36] Seal K. H., Bertenthal D., Samuelson K., Maguen S., Kumar S., Vasterling J. J. (2016). Association between mild traumatic brain injury and mental health problems and self-reported cognitive dysfunction in Iraq and Afghanistan veterans. *Journal of Rehabilitation Research and Development*.

[37] Riley E., Mitko A., Stumps A. (2019). Clinically significant cognitive dysfunction in OEF/OIF/OND veterans: prevalence and clinical associations. *Neuropsychology*.

[38] McGlinchey R. E., Milberg W. P., Fonda J. R., Fortier C. B. (2017). A methodology for assessing deployment trauma and its consequences in OEF/OIF/OND veterans: the TRACTS longitudinal prospective cohort study. *International Journal of Methods in Psychiatric Research*.

[39] Green P. (2004). *Medical Symptom Validity Test (MSVT) for Microsoft Windows: User’s Manual*.

[40] DeGutis J., Agnoli S., Bernstein J. P. K. (2023). Poorer inhibitory control uniquely contributes to greater functional disability in post-9/11 veterans. *Archives of Clinical Neuropsychology*.

[41] Blake D. D., Weathers F. W., Nagy L. M. (1995). The development of a clinician-administered PTSD scale. *Journal of Traumatic Stress*.

[42] Weathers F. W., Keane T. M., Davidson J. R. (2001). Clinician-administered PTSD scale: a review of the first ten years of research. *Depression and Anxiety*.

[43] Lovibond P. F., Lovibond S. H. (1995). The structure of negative emotional states: comparison of the depression anxiety stress scales (DASS) with the Beck depression and anxiety inventories. *Behaviour Research and Therapy*.

[44] First M. B. (1997). *Structured Clinical Interview for DSM-IV Axis I Disorders*.

[45] Melzack R. (1975). The McGill pain questionnaire: major properties and scoring methods. *Pain*.

[46] Buysse D. J., Reynolds C. F., Monk T. H., Berman S. R., Kupfer D. J. (1989). The Pittsburgh sleep quality index: a new instrument for psychiatric practice and research. *Psychiatry Research*.

[47] Fortier C. B., Amick M. M., Grande L. (2014). The Boston Assessment of Traumatic Brain Injury–Lifetime (BAT-L) semistructured Interview. *The Journal of Head Trauma Rehabilitation*.

[48] Federici S., Meloni F., Lo Presti A. (2009). International literature review on WHODAS II (World Health Organization Disability Assessment Schedule II). *Life Span and Disability*.

[49] Bovin M. J., Meyer E. C., Kimbrel N. A. (2019). Using the World Health Organization Disability Assessment Schedule 2.0 to assess disability in veterans with posttraumatic stress disorder. *PLoS One*.

[50] Delis D. C., Kaplan E., Kramer J. H. (2001). Delis-Kaplan Executive Function System. *Assessment*.

[51] De Luca C. R., Wood S. J., Anderson V. (2003). Normative data from the CANTAB. I: development of executive function over the lifespan. *Journal of Clinical and Experimental Neuropsychology*.

[52] Shura R. D., Rowland J. A., Miskey H. M. (2016). Auditory consonant trigrams: a psychometric update. *Archives of Clinical Neuropsychology*.

[53] Woods S. P., Delis D. C., Scott J. C., Kramer J. H., Holdnack J. A. (2006). The California Verbal Learning Test - Second Edition: test-retest reliability, practice effects, and reliable change indices for the standard and alternate forms. *Archives of Clinical Neuropsychology*.

[54] Henry G. K. (2005). Probable malingering and performance on the test of variables of attention. *The Clinical Neuropsychologist*.

[55] Wechsler D. (2008). *Wechsler Adult Intelligence Scale*.

[56] DeGutis J., Chiu C., Thai M., Esterman M., Milberg W., McGlinchey R. (2018). Trauma sequelae are uniquely associated with components of self-reported sleep dysfunction in OEF/OIF/OND veterans. *Behavioral Sleep Medicine*.

[57] Kaur S. S., Gonzales M. M., Eagan D. E., Goudarzi K., Tanaka H., Haley A. P. (2015). Inflammation as a mediator of the relationship between cortical thickness and metabolic syndrome. *Brain Imaging and Behavior*.

[58] Lehmann M., Hagen J., Ettinger U. (2022). Unity and diversity of metacognition. *Journal of Experimental Psychology: General*.

[59] Mazancieux A., Fleming S. M., Souchay C., Moulin C. J. (2020). Is there a G factor for metacognition? Correlations in retrospective metacognitive sensitivity across tasks. *Journal of Experimental Psychology: General*.

[60] Jelinek L., van Quaquebeke N., Moritz S. (2017). Cognitive and metacognitive mechanisms of change in metacognitive training for depression. *Scientific Reports*.

[61] Zhu C., Kwok N. T. K., Chan T. C. W., Chan G. H. K., So S. H. W. (2021). Inflexibility in reasoning: comparisons of cognitive flexibility, explanatory flexibility, and belief flexibility between schizophrenia and major depressive disorder. *Frontiers in Psychiatry*.

[62] Hildenbrand L., Sanchez C. A. (2022). Metacognitive accuracy across cognitive and physical task domains. *Psychonomic Bulletin and Review*.

[63] Sitzmann T., Ely K., Brown K. G., Bauer K. N. (2010). Self-assessment of knowledge: a cognitive learning or affective measure?. *Academy of Management Learning & Education*.

[64] Zell E., Krizan Z. (2014). Do people have insight into their abilities? A metasynthesis. *Perspectives on Psychological Science*.

[65] Zell E., Strickhouser J. E., Sedikides C., Alicke M. D. (2020). The better-than-average effect in comparative self-evaluation: A comprehensive review and meta-analysis. *Psychological Bulletin*.

[66] Moore M. T., Fresco D. M. (2012). Depressive realism: a meta-analytic review. *Clinical Psychology Review*.

[67] Alloy L. B., Abramson L. Y., Alloy L. B. (1988). Depressive Realism: Four Theoretical Perspectives. *Cognitive processes in depression*.

[68] Capobianco L., Heal C., Bright M., Wells A. (2019). What comes first metacognition or negative emotion? A test of temporal precedence. *Frontiers in Psychology*.

[69] Weber F., Exner C. (2013). Metacognitive beliefs and rumination: a longitudinal study. *Cognitive Therapy and Research*.

[70] Huibers M. J. H., Lorenzo-Luaces L., Cuijpers P., Kazantzis N. (2021). On the road to personalized psychotherapy: a research agenda based on cognitive behavior therapy for depression. *Frontiers in Psychiatry*.

[71] Mohlman J., Gorman J. M. (2005). The role of executive functioning in CBT: a pilot study with anxious older adults. *Behaviour Research and Therapy*.

[72] Moritz S., Schneider B. C., Peth J., Arlt S., Jelinek L. (2018). Metacognitive training for depression (D-MCT) reduces false memories in depression. A randomized controlled trial. *European Psychiatry*.

[73] Jelinek L., Hauschildt M., Wittekind C. E., Schneider B. C., Kriston L., Moritz S. (2016). Efficacy of metacognitive training for depression: a randomized controlled trial. *Psychotherapy and Psychosomatics*.

[74] Engeler N. C., Gilbert S. J. (2020). The effect of metacognitive training on confidence and strategic reminder setting. *PLoS ONE*.

[75] Cortese A., Amano K., Koizumi A., Kawato M., Lau H. (2016). Multivoxel neurofeedback selectively modulates confidence without changing perceptual performance. *Nature Communications*.

[76] Hall M. G., Dux P. E. (2020). Training attenuates the influence of sensory uncertainty on confidence estimation. *Attention, Perception & Psychophysics*.

[77] Carpenter J., Sherman M. T., Kievit R. A., Seth A. K., Lau H., Fleming S. M. (2019). Domain-general enhancements of metacognitive ability through adaptive training. *Journal of Experimental Psychology: General*.

[78] Chaytor N., Schmitter-Edgecombe M. (2003). The ecological validity of neuropsychological tests: a review of the literature on everyday cognitive skills. *Neuropsychology Review*.

[79] Horn L., Cimarelli G., Boucherie P. H., Šlipogor V., Bugnyar T. (2022). Beyond the dichotomy between field and lab — the importance of studying cognition in context. *Current Opinion in Behavioral Sciences*.

[80] Schmitter-Edgecombe M., Sumida C., Cook D. J. (2020). Bridging the gap between performance-based assessment and self-reported everyday functioning: an ecological momentary assessment approach. *The Clinical Neuropsychologist*.

