# Supplementary material for: Depressive Symptoms are Associated with More Negative Global Metacognitive Biases in Combat Veterans, and Biases Covary with Symptom Changes over Time
Source: Depress Anxiety. 2023 Apr 17;2023:2925551. doi: 10.1155/2023/2925551 (PMC11921826; doi:10.1155/2023/2925551)
Supplement: Supplementary Materials — For a detailed listing of self-reported and objective cognition measures, see Table S1. For the results of metacognitive sensitivity in health and PTSD, see Figures S1-S2. For the results of metacognitive bias in health by depressive symptoms, see Figure S3. For the changes in metacognitive sensitivity and bias across time points, see Tables S2-4. For correlations between self-reported, objective, and metacognitive bias measures, see Table S5. For the metacognitive biases' relationship with clinical and demographic variables, see Table S6. For the correlations between baseline clinical and demographic variables with changes in self-reported, objective, and metacognitive measures, see Table S7. [file 2925551.f1.docx]

**Supplementary Materials**

**Self-Reported and Objective Cognitive Performance Follow-Up Analyses**

Follow-up group self-report analyses showed that Veterans with only a PTSD diagnosis (*n* = 154) had worse self-reported cognition (*M =* 29.92, *SD* = 17.62) than those with neither disorder (*M =* 21.71, *SD* = 16.03; *t*(300) = 4.24, *p* < .001, FDR-corrected *q* < .001). Similarly, Veterans with only depression (*n* = 19) had worse self-reported cognition (*M = 35.52*, *SD* = 22.45) than those without PTSD or depression (*t*(165) = 3.37, *p* < .001, *q* < .001). Individuals with a comorbid depression and PTSD diagnosis (*n* = 145) had significantly worse self-reported cognitive deficits (*M =* 39.17, *SD* = 19.00) than PTSD alone (*t*(297) = 4.36, *p* = .002, *q* = .002), but not depression (*t*(165) = .64, *p* = .667).

Individuals with a comorbid depression and PTSD diagnosis had significantly higher self-reported health deficits (*M =* 2.53, *SD* = .68) than those without either disorder (*M =* 2.23, *SD* = .64; *t* = -4.02, *p* < .001). However, we observed no significant differences in objective health functioning between those with comorbid disorders (*M =* 1.44, *SD* = 1.30) and without either disorder (*M =* 1.45, *SD* = 1.30; *t* = -.06, *p* = .952). Those with PTSD alone did not show significant differences in self-reported or objective health. However, in depression alone, self-reported health deficits were significantly greater than those without any disorders (*M =* 2.68, *SD* = .75; *t* = -2.94, *p* = .004), and there were no differences in objective health.

**Correlations between PTSD Symptom Clusters and Metacognitive Bias**

We further ran correlations between DSM-IV PTSD symptom clusters and metacognitive bias in order to see if avoidance and numbing symptoms were more related to metacognitive bias than re-experiencing or hyperarousal symptoms, given that the former are more related to depression. While avoidance and numbing symptoms were more associated with depressive symptoms (*ρ* = .58, *p* < .001) than re-experiencing and hyperarousal symptoms (*ρ’s* = .43, .38; *p’s* < .001, respectively), they were similarly associated with metacognitive bias (*ρ* = -0.20, *p* < .001) as re-experiencing or hyperarousal symptoms (*ρ* = -0.19, -.21; *p’s* < .001, respectively).

**Associations between Changes in PTSD and Depression with Changes in Global Metacognition and Health Awareness**

In order to more completely characterize changes in metacognition, we separated participants into 2 separate groups of 2 (i.e., for depression, improving (depression time 1, no depression at time 2; *n* = 42) and worsening depression (no depression at time 1, depression at time 2; *n* = 25), as well as improving PTSD (*n* = 46) and worsening PTSD (*n* = 21). For depression diagnosis changes, we ran a 2 (improving/worsening) x 2 (time 1/time 2) repeated-measures ANOVA on metacognitive bias and found a significant interaction (F = 4.16, *p* = .046, see Table S4), such that changes in metacognitive bias were associated with Veterans’ depression diagnoses between timepoints. For PTSD diagnosis changes, we ran a 2 (improving/worsening) x 2 (time 1/time 2) repeated-measures ANOVA on metacognitive bias and similarly found a significant interaction (F = 10.55, *p* = .002). The difference in metacognitive bias across timepoints was associated with the difference in depression (*ρ* = -.245, *p* < .001) and PTSD (*ρ* = -.329, *p* < .001), such that decreases in PTSD and depressive symptoms were associated with more positive metacognitive bias.

Generally speaking, while self-reported cognition fluctuated across groups, objective cognition did not, suggesting that changes in metacognition across timepoints were mediated by changes in self-reported cognition. Veterans that no longer met criteria for depression at time 2 (*n* = 40) saw significant improvements in their self-reported cognition (T1 *M* = 35.32, *SD* = 19.70; T2 *M* = 29.17, *SD* = 18.75; *t* = 2.20, *p* = .033), but not objective cognition or metacognitive bias. Conversely, Veterans that developed a depression at time 2 (*n* = 25) saw nonsignificant but numerical reductions in self-reported cognition (T1 *M* = 34.00, *SD* = 21.97; T2 *M* = 40.33, *SD* = 18.03; *t* = -1.56, *p* = .132) and increased negative metacognitive bias (T1 *M* = .13, *SD* = 1.42; T2 *M* = -.41, *SD* = 1.31; *t* = 1.79, *p* = .087). Paradoxically, in Veterans who developed depression, metacognitive sensitivity scores numerically improved (T1 *ρ* = -.02, *p* = .934; T2 *ρ* = -.19, *p* = .369) and worsened in those who no longer had depression at time 2 (T1 *ρ* = -.27, *p* = .081; T2 *ρ* = -.17, *p* = .301).

Veterans with improved PTSD (*n* = 46) saw marginally significant *reductions* in objective cognitive performance (T1 *M* = .03, *SD* = .44; T2 *M* = -.13, *SD* = .56; *t* = 1.95 , *p* = .057), despite improvements in both self-reported cognition (T1 *M* = .29.71, *SD* = 18.46; T2 *M* = 19.20, *SD* = 15.07; *t* = 5.01, *p* < .001), and metacognitive bias (T1 *M* = -.08, *SD* = 1.24; T2 *M* = .63, *SD* = .1.23; *t* = -3.89, *p* < .001). This might be due to the sampling bias in improved PTSD, where 13/46 had depression at time 1, and 5/46 at time 2. Veterans who no longer had PTSD at time 2 (*n* = 21) had no significant differences across self-reported, objective, and metacognitive bias measures. There were no changes in metacognitive sensitivity between timepoints in Veterans with PTSD. For the full results, see Table S1.

Similar to the results with cognition, differences in metacognitive health bias across time were associated with differences in depression (*ρ* = -.14, *p* = .021) and PTSD (*ρ* = -.13, *p* = .039), such that decreases in depression and PTSD symptoms were associated with more positive metacognitive bias. These differences were led by differences in self-reported health with changes in both depressive (*ρ* = -.14, *p* < .001) and PTSD symptoms (*ρ* = -.17, *p* < .001), while changes in objective health were not significant. Overall, self-reported, objective, and metacognitive biases did not significantly differ between timepoints. Further separating between groups revealed numerical decreases in metacognitive accuracy in those who no longer had depression at time 2 (T1 *ρ* = .35, *p* = .023; T2 *ρ* = .15, *p* = .355; See Table S2), which was notably consistent with the cognition results. In contrast, we observed numerical improvement for individuals with improved PTSD in metacognitive health accuracy.

**Global Metacognitive Sensitivity vs. Bias in the Extended Sample**

We replicated the key continuous analyses in our Veteran sample while including individuals who reported no significant interference on their daily life. This included a total sample of 618 Veterans. As before, we examined metacognitive sensitivity in cognition (*ρ* = -0.21, *p* < .001) which was not significantly different from our interference sample. We next examined metacognitive bias in awareness and found that a more negative metacognitive bias was significantly associated with increased depressive (*ρ* = -.41, *p* < .001) and PTSD (*ρ* = -.31, *p* < .001) symptoms. Notably different than before, both depressive and PTSD symptoms were significantly related to metacognitive bias in a joint model (depression: *β* = -.30, *p* < .001; PTSD: *β* = -.13, *p* = .005), though depressive symptoms clearly had a greater independent effect. Depression similarly mediated PTSD in predicting metacognitive bias, such that worse depressive symptoms partially explained the relationship between PTSD symptoms and more negative metacognitive bias (*β* = -.22, 95% CI (-.30, -.14)) with a direct effect of PTSD remaining significant (*β* = -.16, *p* = .005).

Metacognitive sensitivity in health awareness was similar to our interference sample (*ρ* = .22, *p* < .00). An association was found between negative metacognitive bias and increased depressive (*ρ* = -.19, *p* < .001) and PTSD (*ρ* = -.15, *p* < .001) symptoms. Both depressive and PTSD symptoms were related to metacognitive health bias in a joint model (depression: *β* = -.10, *p* = .048; PTSD: *β* = -.13, *p* = .008). PTSD also mediated depressive symptoms in predicting metacognitive health bias, such that worse PTSD symptoms partially explained the relationship between depressive symptoms and more negative metacognitive health bias (*β* = -.10, 95% CI (-.17, -.02)) with a direct effect of depression remaining significant (*β* = -.12, *p* = .048). Depressive symptoms did not mediate the role of PTSD symptoms in metacognitive health bias.

**Associations between Changes in Clinical Variables and Changes in Metacognitive Sensitivity and Bias in the Extended Sample**

We replicated whether metacognitive measures changed with changes in PTSD and depressive symptoms over an approximately 2-year period in the extended sample. Changes in metacognitive bias were associated with changes in both depressive (*ρ* = -.24, *p* < .001) and PTSD (*ρ* = -.32, *p* < .001) symptoms, such that decreases in PTSD and depressive symptoms were associated with more positive increases in metacognitive bias. In a joint regression, the difference between PTSD symptoms and depressive symptoms across timepoints explained unique variance in metacognitive bias changes (R² = .12; *β* = -.24, *p* < .001; *β* = -.18; *p* < .001, respectively). PTSD also mediated depressive symptoms in predicting changes in metacognitive bias, such that worse PTSD symptoms partially explained the relationship between depressive symptoms and more negative metacognitive bias (*β* = -.12, 95% CI (-.19, -0.05)) with a direct effect of depression remaining significant (*β* = -.25, *p* < .001).

Changes in metacognitive health bias were not significantly related to PTSD or depression.

**Figure S1**

*Metacognitive Health Sensitivity by Depression (150 bin)*

**
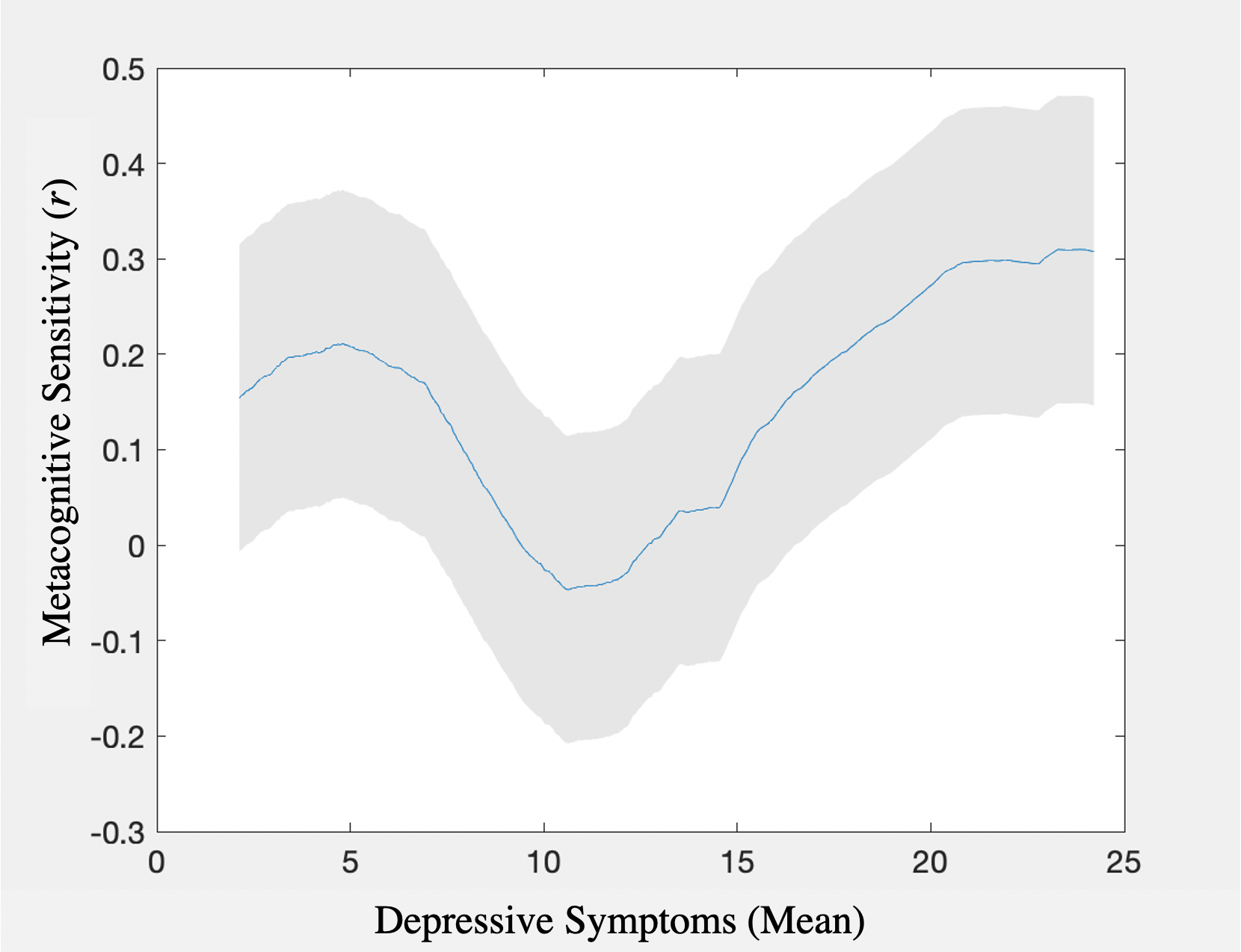
**

*Note.* Metacognitive Sensitivity is calculated as the correlation between self-reported functioning on the WHODAS II and objective cognitive functioning on a battery of cognitive tasks. Sensitivity was graphed using a continuous sliding window by calculating 100 iterations of sensitivity at respective depressive symptoms using bins of n = 150.

**Figure S2**

*Metacognitive Sensitivity by PTSD (150 bin)*

**
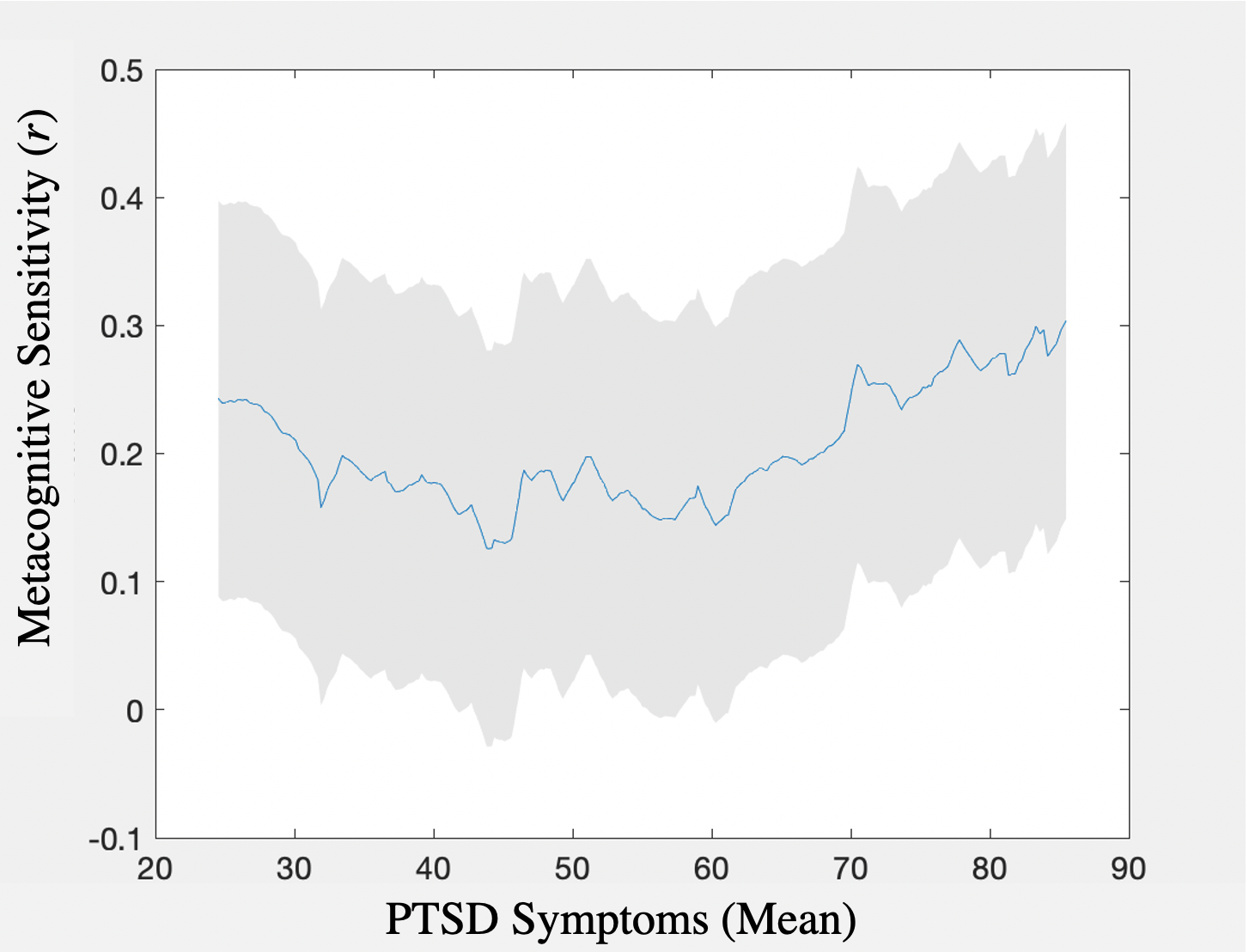
**

*Note.* Metacognitive Sensitivity was calculated as the correlation between self-reported functioning on the WHODAS II and objective cognitive functioning on a battery of cognitive tasks. Sensitivity was graphed using a continuous sliding window by calculating 100 iterations of sensitivity at respective PTSD symptoms using bins of n = 150.

**Figure S3**

*Scatterplot of Metacognitive Health Bias by Depressive Symptoms*


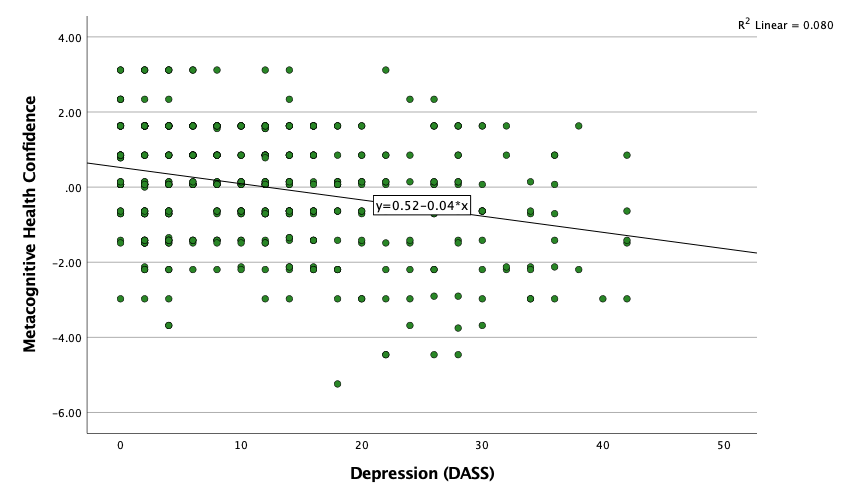


*Note.* Higher Metacognitive bias indicates more positive judgements of one’s health relative to our sample. Greater DASS depression scores indicate more severe depressive symptoms.

**Table S1**

1. *Self-Reported Cognition and Health Measures*

**
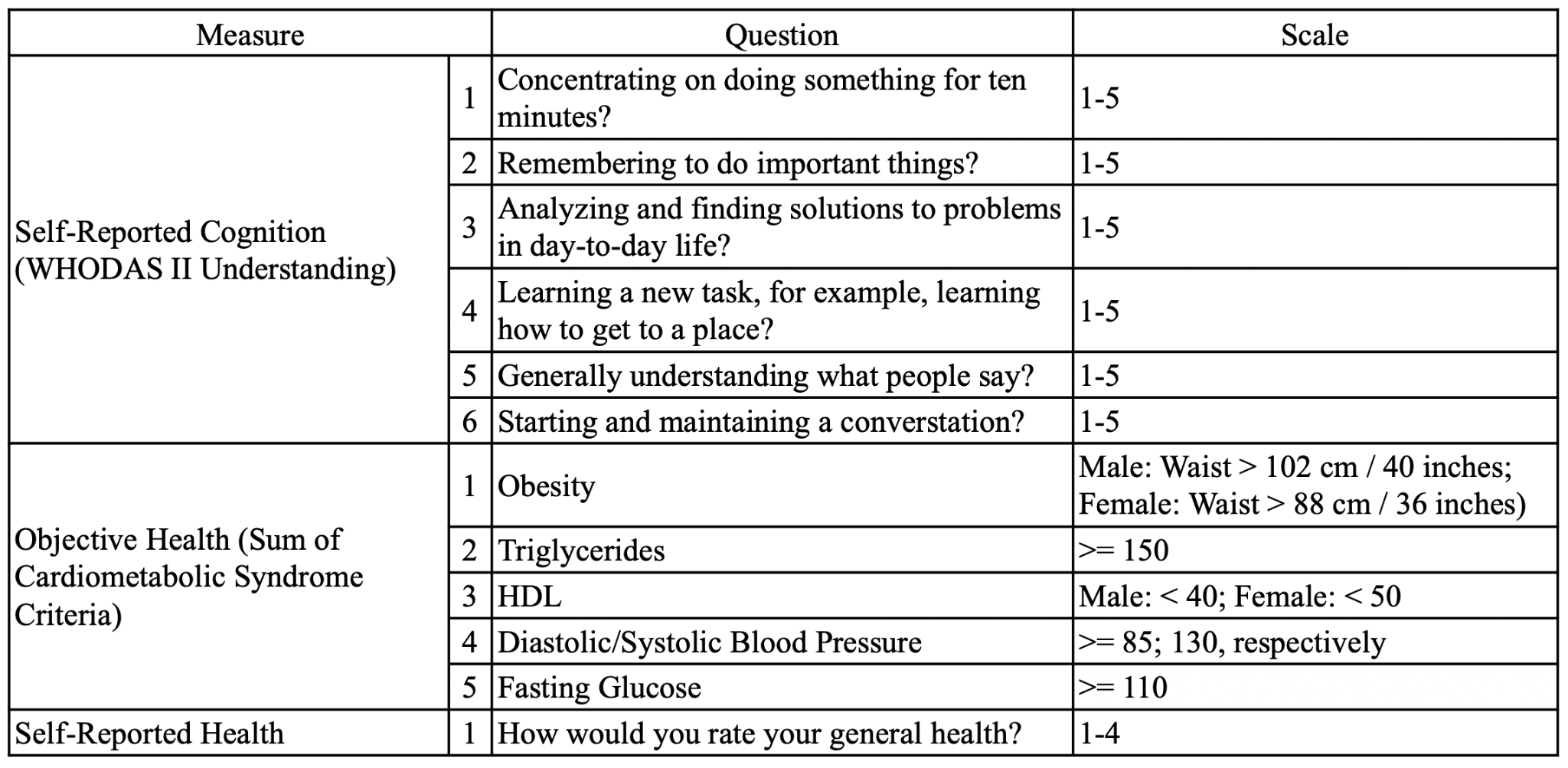
**

1. *Objective Cognition Measures
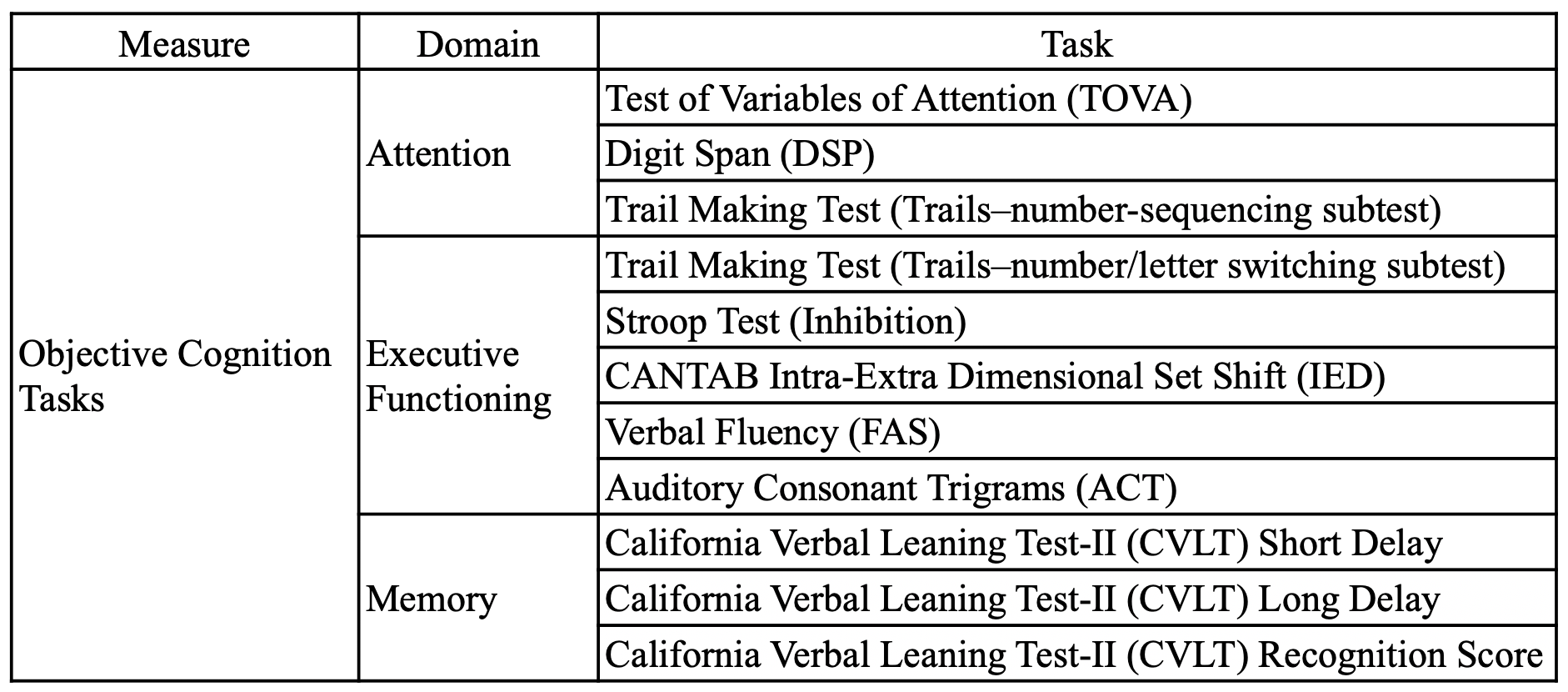
*

**Table S2**

*Changes in Metacognitive Sensitivity and Bias Across Depression* *and PTSD Groups*


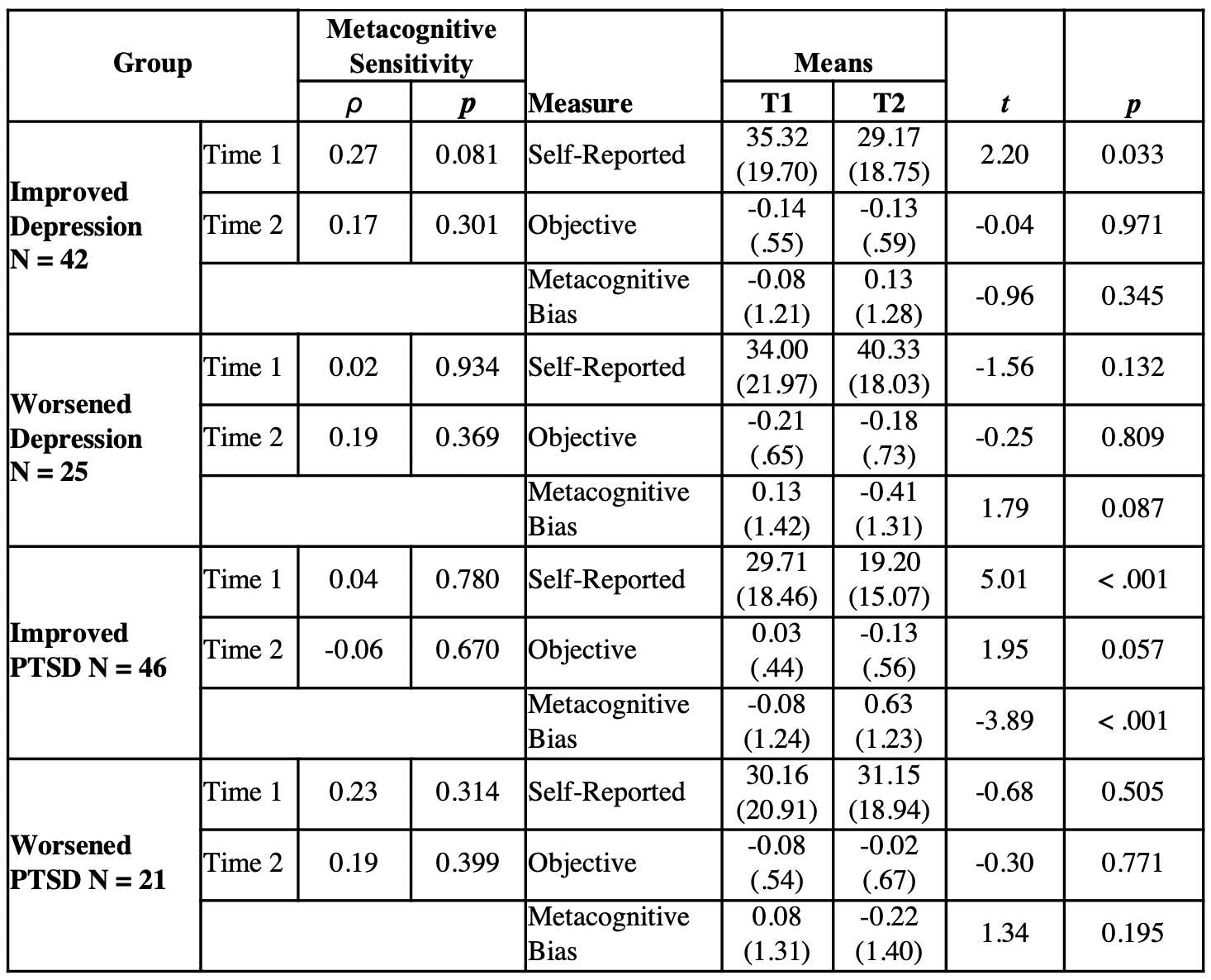


*Note.* **p* < .05; ***p* < .01

**Table S3**

*Changes in Metacognitive Health Sensitivity and Bias Across* depression *and PTSD Groups*


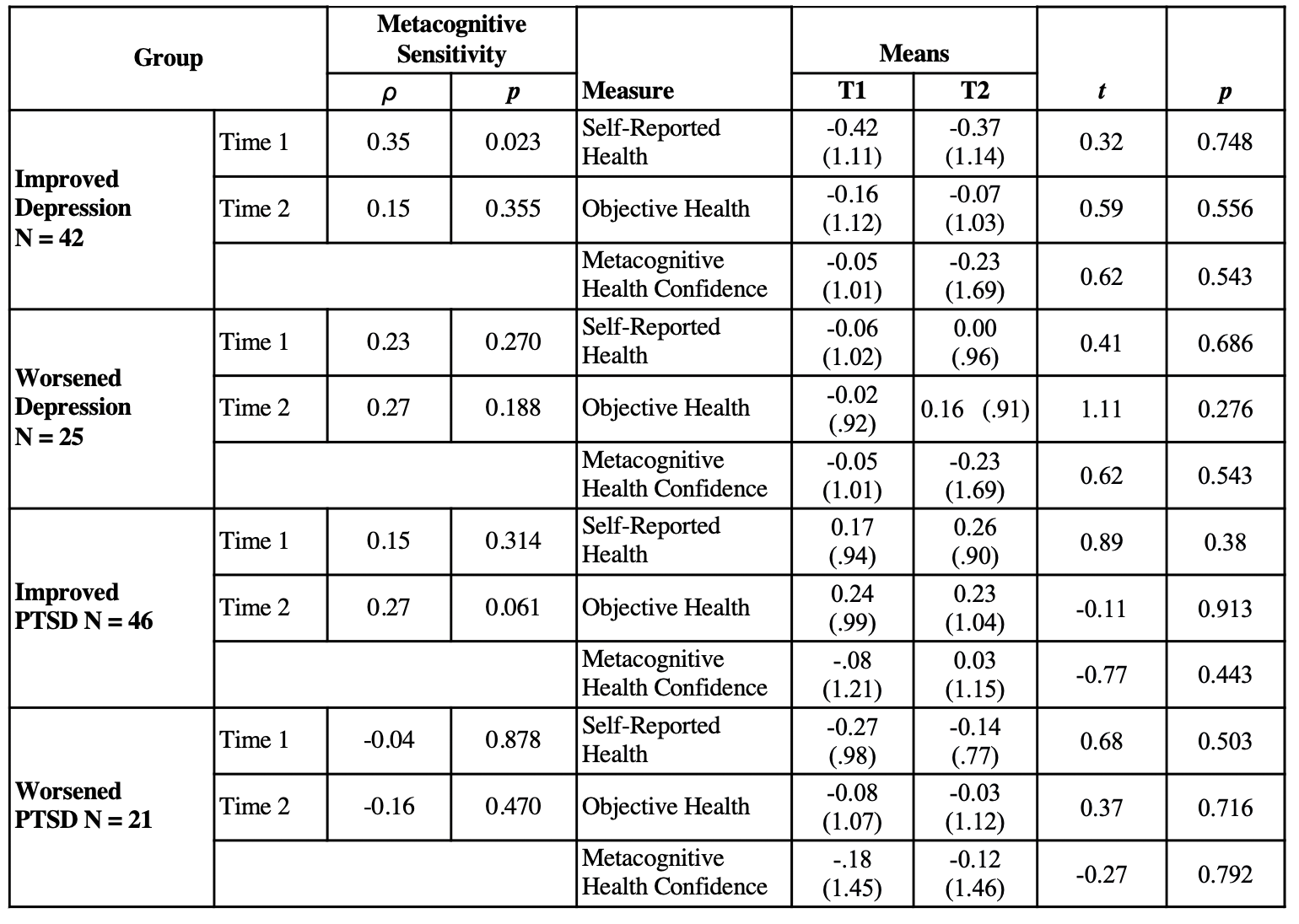


*Note.* **p* < .05; ***p* < .01

**Table S4**

*Repeated Measures Diagnosis Change by Measure Interaction*


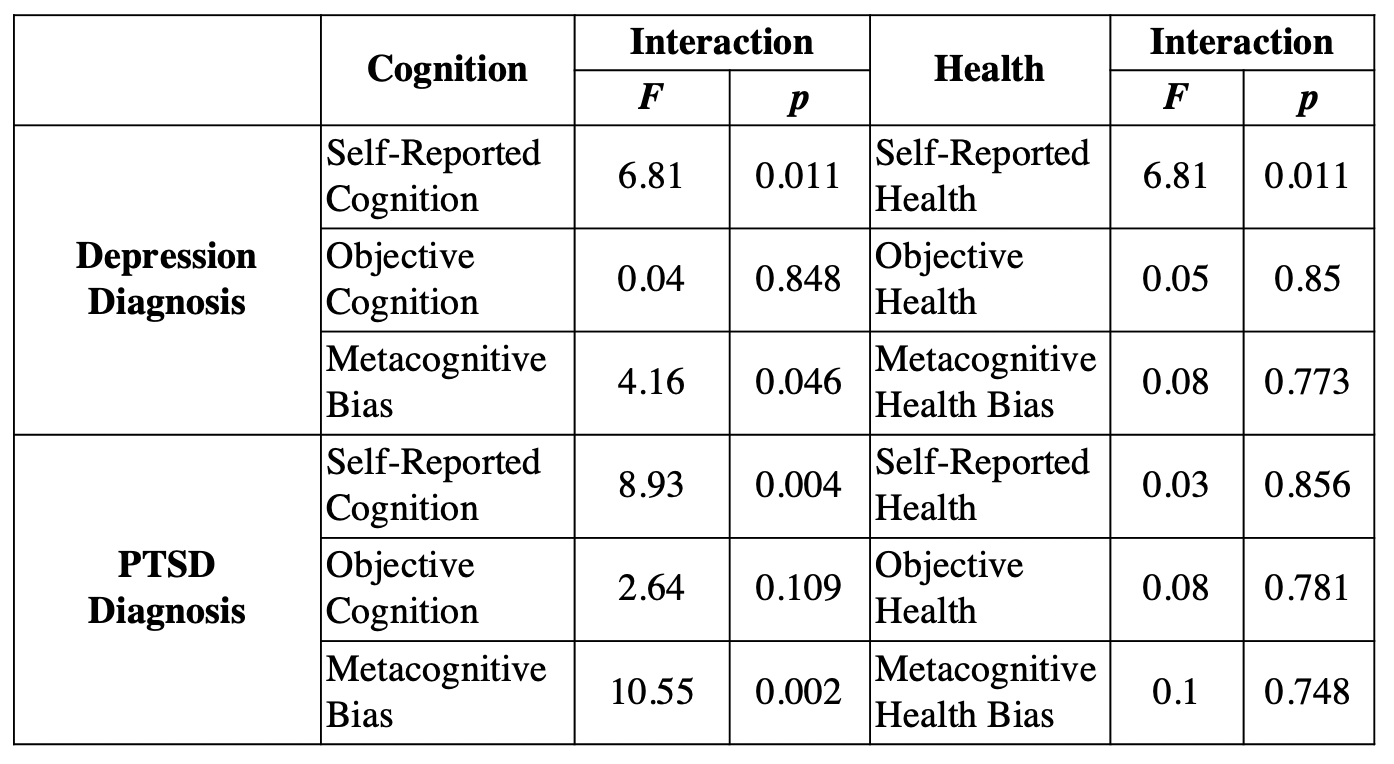


*Note.* Within-samples repeated measures ANOVA was run to identify group changes (improved to worsening PTSD or depression diagnoses, respectively) as between-subjects factor and within-subjects cognition or health measure as repeated measure. Interaction effects were calculated to observe how changes in diagnoses were related to respective measures.

**Table S5**

*Correlations between Self-Reported, Objective, and Metacognition Measures with Variables of Interest Across Sample*


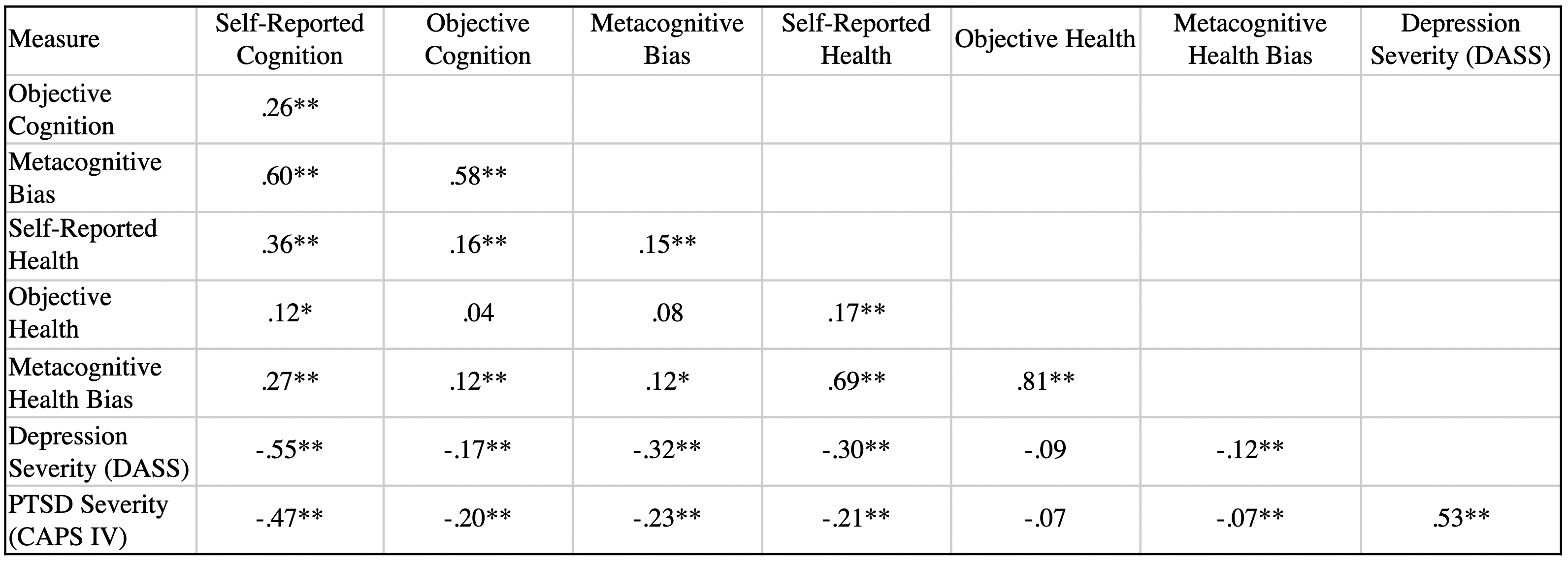


*Note.* **p* < .05; ***p* < .01

**Table S6**

*Metacognitive Bias Correlations with Clinical and Demographic Variables*


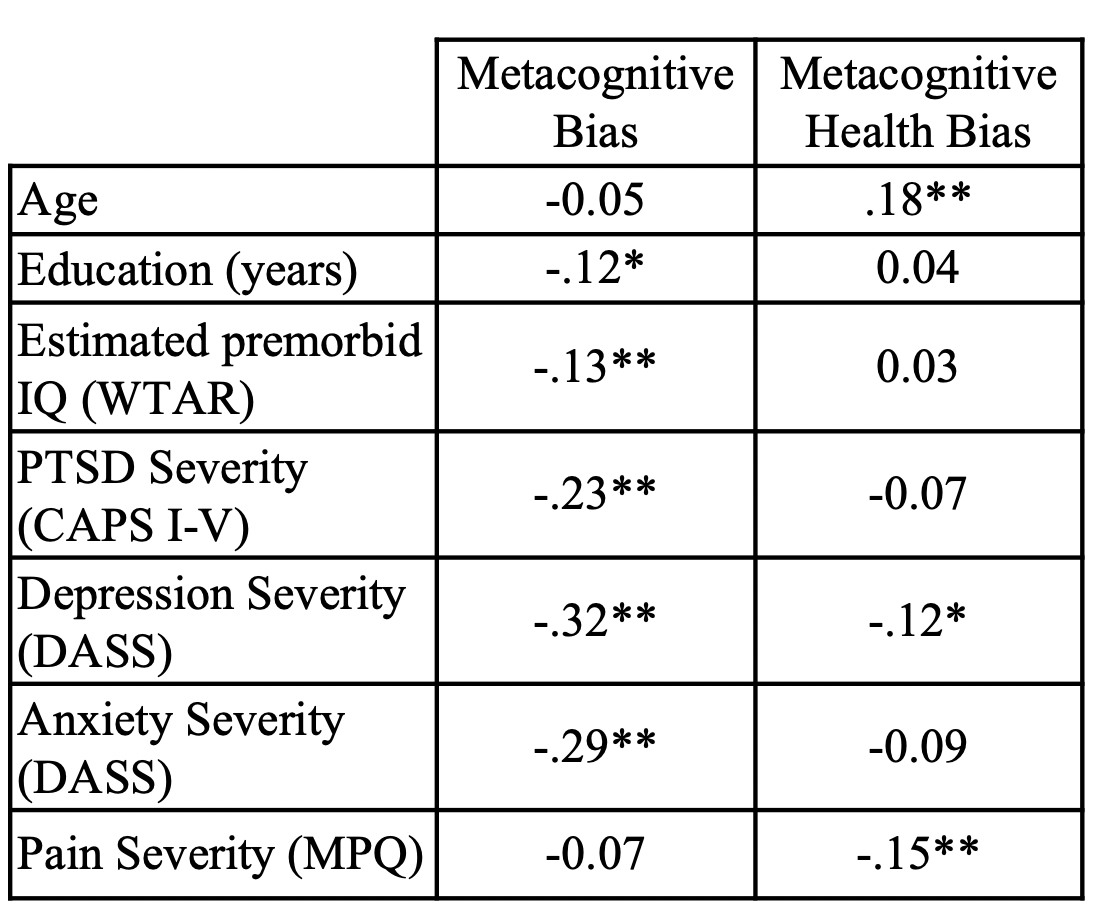


*Note.* **p* < .05; ***p* < .01

**Table S7**

*Correlations Between Baseline Clinical and Demographic Variables with T1-T2 Difference Scores in Self-Reported, Objective, and Metacognitive Cognition and Health Measures*


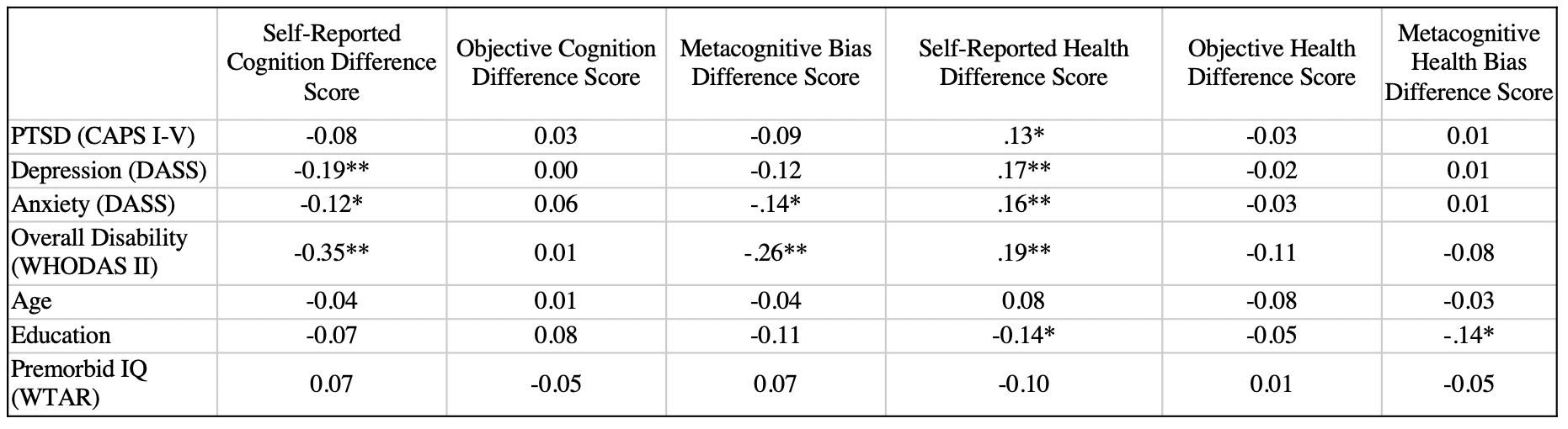


*Note.* **p* < .05; ***p* < .01
